# Human forager response to abrupt climate change at 8.2 ka on the Atlantic coast of Europe

**DOI:** 10.1038/s41598-022-10135-w

**Published:** 2022-05-02

**Authors:** Asier García-Escárzaga, Igor Gutiérrez-Zugasti, Ana B. Marín-Arroyo, Ricardo Fernandes, Sara Núñez de la Fuente, David Cuenca-Solana, Eneko Iriarte, Carlos Simões, Javier Martín-Chivelet, Manuel R. González-Morales, Patrick Roberts

**Affiliations:** 1 Department of Archaeology, Max Planck Institute for the Science of Human History, Jena, Germany; 2 Departamento de Ciencias Humanas, Universidad de La Rioja, Logroño, Spain; 3 Department of Prehistory and Institute of Environmental Science and Technology (ICTA), Universitat Autònoma de Barcelona, Bellaterra, Spain; 4 Instituto Internacional de Investigaciones Prehistóricas de Cantabria, Universidad de Cantabria, Santander, Gobierno de Cantabria, Santander, Spain; 5 Grupo de I+D+I EVOADAPTA, Departamento de Ciencias Históricas, Universidad de Cantabria, Santander, Spain; 6 Department of Archaeology, University of Cambridge, Downing Street, Cambridge, CB2 3DZ UK; 7 School of Archaeology, University of Oxford, Oxford, UK; 8 Faculty of Arts, Masaryk University, Brno, Czech Republic; 9 Centre de Recherche en Archéologie, Archeosciences, Histoire (CReAAH), UMR-6566, Université de Rennes 1, Rennes, France; 10 Laboratorio de Evolución Humana-IsoTOPIK Stable Isotope Laboratory, Universidad de Burgos, Burgos, Spain; 11 ICArEHB—Interdisciplinary Center for Archaeology and the Evolution of Human Behaviour, Universidade Do Algarve, Faro, Portugal; 12 Facultad de Ciencias Geológicas, Universidad Complutense de Madrid & Instituto de Geociencias (CSIC-UCM), Madrid, Spain; 13 School of Social Sciences, University of Queensland, Queensland, Australia

**Keywords:** Palaeoclimate, Behavioural ecology, Archaeology

## Abstract

The cooling and drying associated with the so-called ‘8.2 ka event’ have long been hypothesized as having sweeping implications for human societies in the Early Holocene, including some of the last Mesolithic hunter-gatherers in Atlantic Europe. Nevertheless, detailed ‘on-site’ records with which the impacts of broader climate changes on human-relevant environments can be explored have been lacking. Here, we reconstruct sea surface temperatures (SST) from δ^18^O values measured on subfossil topshells *Phorcus lineatus* exploited by the Mesolithic human groups that lived at El Mazo cave (N Spain) between 9 and 7.4 ka. Bayesian modelling of 65 radiocarbon dates, in combination with this δ^18^O data, provide a high-resolution seasonal record of SST, revealing that colder SST during the 8.2 ka event led to changes in the availability of different shellfish species. Intensification in the exploitation of molluscs by humans indicates demographic growth in these Atlantic coastal settings which acted as refugia during this cold event.

## Introduction

Current global climate warming is having, and will continue to have, widespread consequences for humans. Looking to the past, multiple climatic and environmental changes have long been thought to have shaped human evolution and behaviour^1–3^. The Holocene (11.7–0 ka cal BP) is a geological epoch characterized by comparative stable climatic conditions. However, that stability was punctuated by a series of sudden climate changes, particularly during the Early Holocene^4^. Among these, the ‘8.2 ka event’ has been identified as the largest and most abrupt climatic event of the Holocene^5,6^. Climate scientists suggest that this ‘event’ was the result of an outburst of glacial meltwater from the Laurentide lakes in North America^7^. The influx of cold water into the Atlantic Ocean led to a reduction of sea surface salinity and a decline of the Atlantic meridional overturning circulation (AMOC), provoking a reduction in sea surface temperatures (SST) across the North Atlantic^8^. The cooling effects of this event have been documented in proxies from the Greenland ice cores^6^ and across Europe^5,9,10^. Short and sharp periods of colder or drier conditions have also been recorded ~ 8.2 ka throughout the northern^11–13^ and southern hemispheres^11,12,14^. Nevertheless, while now a well-established climatic phenomenon, the sweeping impacts of the ‘8.2 ka event’ on different environments often remain assumed rather than proven, and local records of marine or terrestrial conditions available at an appropriate resolution often remain lacking. Furthermore, while temperature or rainfall changes associated with the ‘8.2 ka event’ are often well-established, there are few palaeoclimatic proxies available that have enabled insights into the influence of the ‘8.2 ka event’ on the seasonality of climatic conditions in different parts of the world.

These gaps relating to the impact of this cold event on local environments are problematic given that there have been a number of attempts to link the ‘8.2 ka event’ to changes in past human societies, with cooling and drying being argued to have stimulated behavioural or settlement responses^15,16^. In the Middle East, a 300-year period of drying and cooling has been associated with economic and cultural changes leading to an increase in social stratification and urbanism in the region^17^. Aridity at ~ 8.2 ka has also been correlated with changes in settlement patterns and human subsistence in North and East Africa^18^. On the Atlantic façade of Europe, the ‘8.2 ka event’ occurred at a time when some of the last Mesolithic hunter-gatherers in Europe were producing dense shell middens along the coast prior to the arrival of agriculture^19^. Given the apparent reliance of human societies on marine resources, it has been hypothesised that this abrupt climate change must have affected the availability of exploited marine taxa and thus the foraging behaviours, settlement patterns, and spatial mobility of prehistoric human populations^20^. However, as with previous examples, high-resolution local palaeoclimatic records that are directly associated with areas of human occupation have remained generally lacking^16^. Moreover, given the known importance of seasonal patterns in subsistence strategies to hunter-gatherers^21,22^, seasonal records of changes in marine and terrestrial conditions are particularly important. Nonetheless, suitable proxies and precise sequence chronologies have remained scarce.

Marine molluscs have very specific environmental requirements and are extremely sensitive to climate change, as revealed by previous studies across Atlantic Europe^23,24^. Recent research has demonstrated that the current rising of SST is forcing a rapid expansion of the northern limit of warm-adapted marine species^24,25^. Similarly, warmer conditions during the Early Holocene have been argued to have led to significant changes in species representation when compared to Pleistocene glacial conditions^26^. Mollusc species recovered from archaeological deposits thus have high potential to provide temperature information of direct significance to humans occupying particular sites and exploiting marine resources^27^. Recent developments in the radiocarbon dating of shell carbonate have improved our understanding of the dating of this material, allowing researchers to accurately determine the chronology of climate change decoded from shell remains^28^. Beyond taxonomic indications, stable oxygen isotope ratios derived from archaeologically-recovered marine mollusc shells (δ^18^O_shell_) have been demonstrated to be powerful recorders of the seasonal SST variations experienced by that animal in the past^29,30^, offering unparalleled insights into the seasonality of marine environments relied upon by past human populations. Significantly, the effectiveness of these seasonal palaeothermometers has also been demonstrated on the European Atlantic façade for *Phorcus lineatus* (da Costa, 1778)^21,31,32^, a topshell species that comprises a significant proportion of Mesolithic shell middens found in the region. The difficult task, until now, however, has been to find a shell midden with sufficient temporal resolution to enable inference of short and abrupt changes in climate conditions and human behaviour^19^.

**Figure 1:**
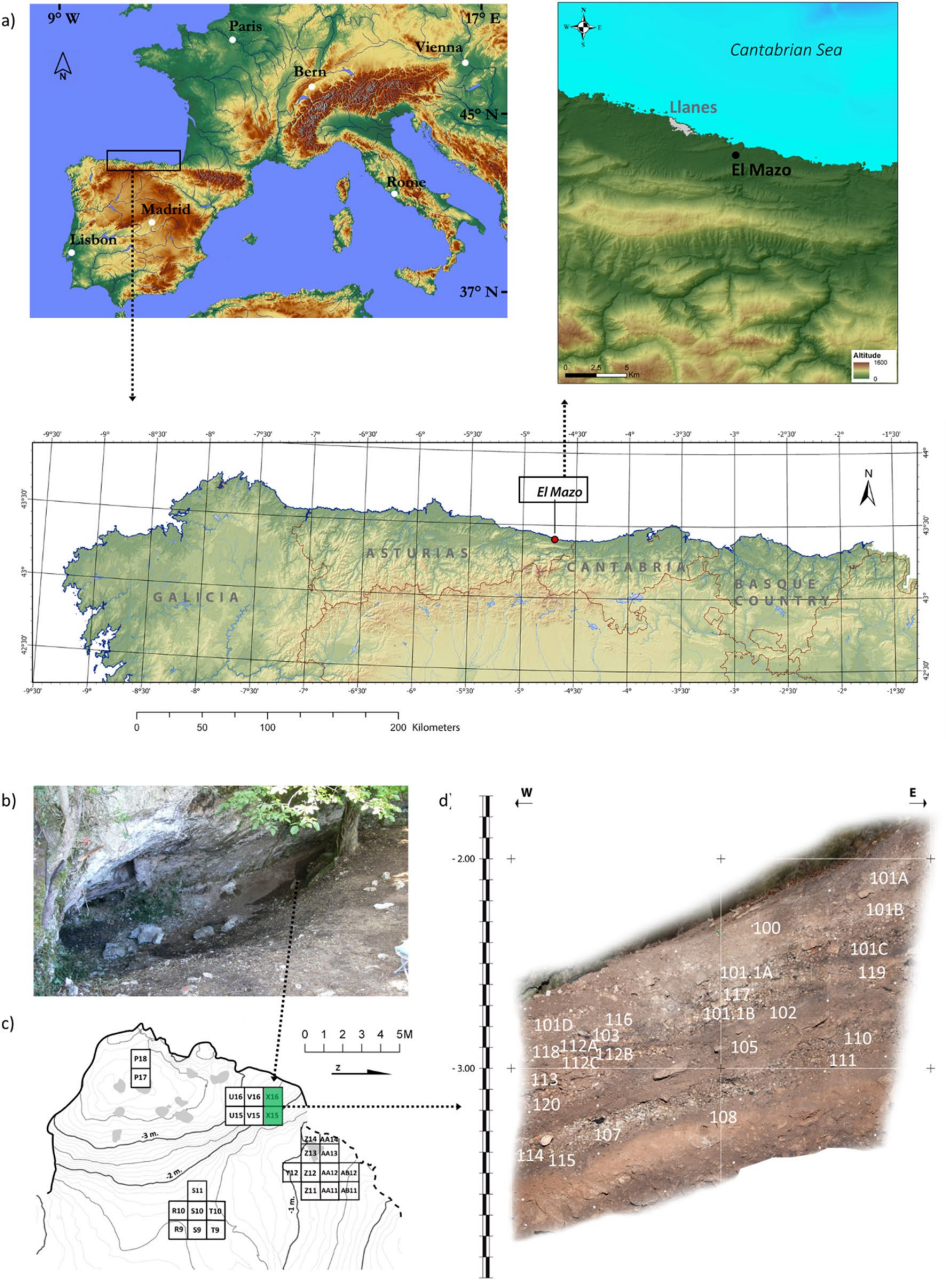
(**a**) Location of the study area (Cantabrian region, northern Spain) and the shell midden site of El Mazo. (**b**) External view of El Mazo; (**c**) topographic map of the site showing excavation areas and provenance of the studied samples (shaded squares), and (**d**) stratigraphy of the inner test pit (squares X15 and X16). Satellite map in the upper-left corner of (**a**) was created by authors using Adobe® Illustrator software. The original base map was extracted from the maps-for-free web site (https://maps-for-free.com/) (© Collaborators of OpenStreetMap, Open Database License [ODbL]). Map in the upper-right corner of (**a**) was generated by Alejandro Garcia-Moreno using ArcGIS software. Lower map of (**a**) was created by Luis C. Teira Mayolini using ArcGIS and Adobe Suite software.

Here, we report chronological, archaeomalacological and isotopic data from shellfish remains recovered from the site of El Mazo in northern Spain (Fig. 1; Supplementary Text 1; Supplementary Fig. 1). We performed a large number of radiocarbon measurements on different materials, including bone from terrestrial animals, microbotanical remains, and marine mollusc shells to generate a high-resolution chronological sequence of the human use of the cave. Meanwhile, measurements of δ^18^O_shell_ on subfossil topshells *P. lineatus* were used to accurately reconstruct short-term changes in coastal SST throughout the Early Holocene. In addition, distributions of shell assemblages were quantified to determine marine mollusc species representation, diachronic changes in shell size, harvesting areas, and the occurrence of shell size selection by humans. Significantly, the site is unique in the context of the European Atlantic façade given its long stratigraphic sequence (9–7.4 ka cal BP), but especially, the high chronological resolution of each unit. As a result, it offers an important opportunity to decipher, for the first time, the duration and the seasonal impact of the 8.2 ka event on SST in Atlantic Europe, its impacts on littoral resources that were being heavily exploited by humans, and its broader ramifications for the settlement patterns and economies of some of the last dedicated hunter-gatherer groups in Europe prior to the expansion of domesticated plants and animals into the region. The study of ‘on-site’ palaeoenvironmental indicators, at a seasonal resolution, also demonstrates the significant potential of this methodology, which can be applied to multiple sites, to explore the overall, and intra-annual, effects of abrupt climate changes on specific environments where the resolution of archaeological sites allows.

## Results

### Bayesian chronological modelling of El Mazo

**Figure 2:**
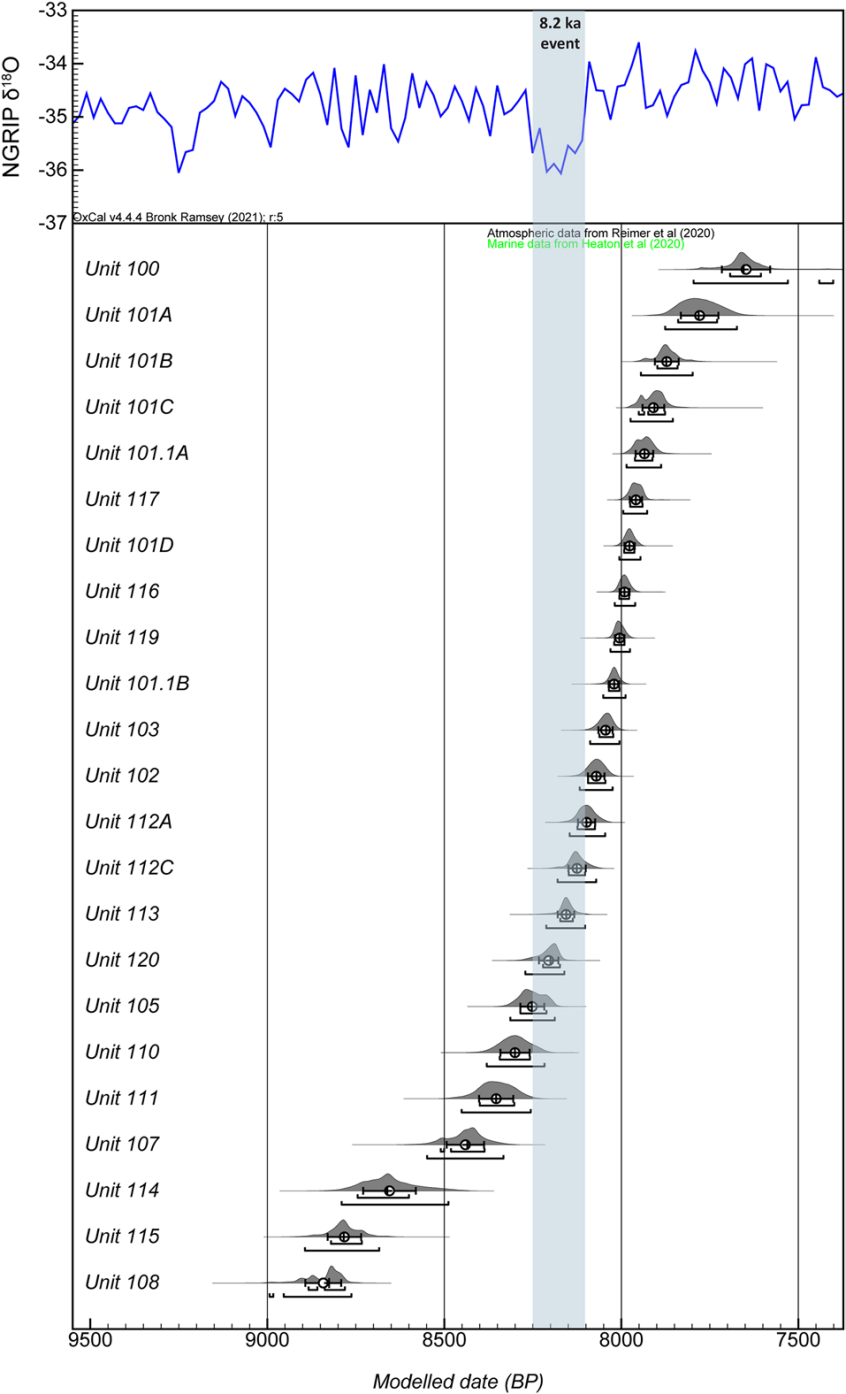
Radiocarbon-based chronology for the midden site sequence of El Mazo cave (Asturias, N Spain) modelled using OxCal v.4.4.2^33,34^ and the radiocarbon calibration curves IntCal20^35^ and Marine20^36^. Sequence of posterior distributions summarize the chronology of archaeological phases at El Mazo. For each archaeological phase, the OxCal Date command for an undated object was used to summarize the chronology of a phase. The full output of the Bayesian chronological model, showing estimates for start and end of archaeological phases, is provided in Supplementary Data 1.

Since 2012, a total of 65 ^14^C AMS measurements were obtained for archaeological samples from 23 of the 25 stratigraphic units documented in squares X15 and X16 of the shell midden site of El Mazo (Fig. 1c; Supplementary Text 1; Supplementary Table 1; Supplementary Table 2): 18 on bones from terrestrial mammals, specifically ungulates with anthropogenic modifications; 5 on wood charcoal; and 42 on marine shells. 57 ^14^C measurements were produced at the Oxford Radiocarbon Accelerator Unit (ORAU) and 8 at the International Chemical Analyses (ICA) laboratory (Supplementary Table 1; Supplementary Table 2). The Bayesian modelled sequence places the shell midden formation in the Early and Middle Holocene, between 9.0 and 7.4 ka cal BP (Fig. 2; Supplementary Fig. 2; Supplementary Data 1). Bayesian modelling offers a high chronological resolution, allowing for the identification of a unit which formed precisely during the 8.2 ka event (Fig. 2). Three out of thirteen shells used for δ^18^O analysis to reconstruct SST, and that were radiocarbon dated, were identified as outliers by the Bayesian model (Supplementary Data 1) and were thus discarded from the palaeoclimatic discussion (Supplementary Text 2).

### Coastal sea surface temperatures (SST) during the early Holocene

**Figure 3:**
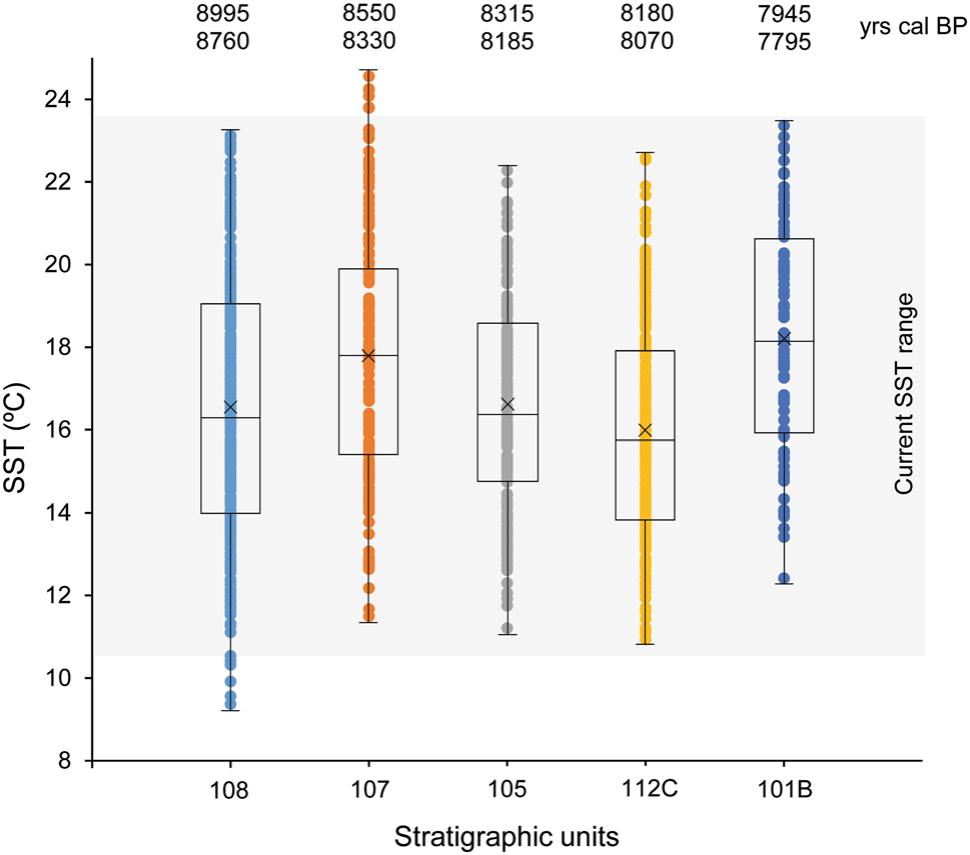
Estimated sea surface temperature (SST) derived from δ^18^O_shell_ values obtained from *P. lineatus* shells recovered from the shell midden site of El Mazo cave (Asturias, N Spain). SST was calculated using Eqs. (1) and (2). Grey square shows current SST range in N Spain^31,32^.

SST derived from δ^18^O_shell_ values indicates important changes in absolute values for both annual maxima and minima, as well as in the SST annual range over time (Fig. 3; Supplementary Data 2). The coldest winter temperature (9.4 °C) and largest seasonality (i.e., difference between winter and summer) (13.8 °C) were recorded at the bottom of the sequence (unit 108: 8995–8760 cal BP). Minimum and maximum SST increased at unit 107 (8550–8330 cal BP) by 2.1 and 1.4 °C, respectively. Seasonality during this period (13.1 °C) was lower than in the oldest unit. For both units dating to the 8.2 ka event (105: 8315–8185 cal BP; 112C: 8180–8070 cal BP) there was a decrease in the minimum temperature in comparison with the three centuries before and, specifically, a reduction of 2.3 °C in summer temperatures. These were the coldest summer temperatures of the entire sequence (22.3–22.6 °C). Seasonality decreased, showing the smallest range of the entire sequence (11.1–11.7 °C). SST then increased in the upper part of the sequence (101B: 7945–7795 cal BP). During this period, the warmest temperature in winter (12.4 ºC) was recorded, reporting even slightly higher winter SSTs than today in the region.

### Marine mollusc species representation and climate changes

**Figure 4:**
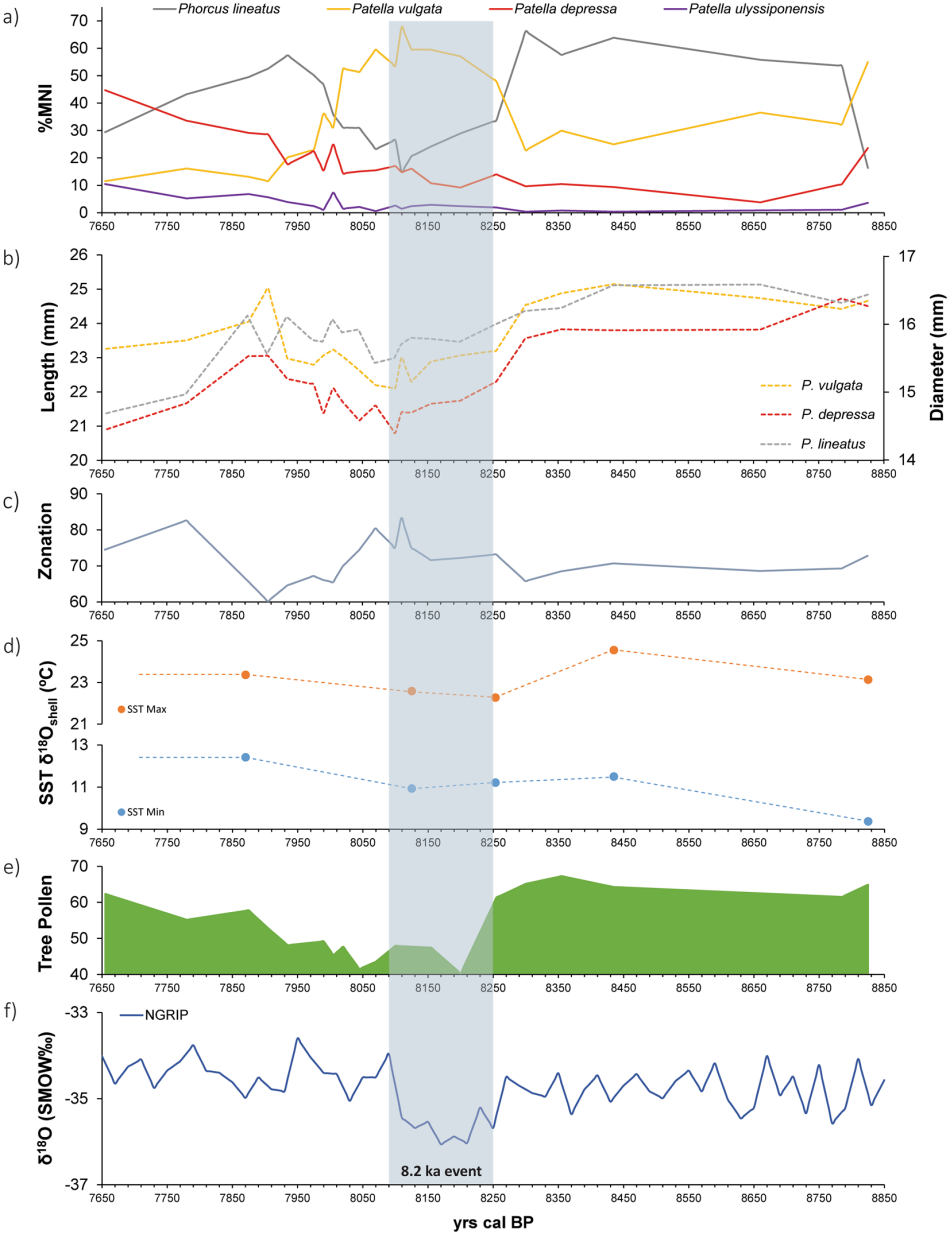
(**a**) Relative representation of the four main marine mollusc taxa recovered from El Mazo site over time (*P. lineatus*, *P. vulgata*, *P. depressa*, and *P. ulyssiponensis*). (**b**) Average shell size variations on *P. lineatus*, *P. vulgata* and *P. depressa* through time. (**c**) Percentage of *P. vulgata* shells collected in the lower intertidal zone over time deduced from L/H ratios^37^. (**d**) Maximum and minimum estimated SST from δ^18^O_shell_ values for different stratigraphic units through shell midden sequence. (**e**) Percentage of arboreal pollen over time in N Spain^38^. (**f**) δ^18^O from the Greenland NGRIP ice core^39^. Blue bar shows 8.2 k event duration according to the Greenland NGRIP ice core^6^.

From the study of the shell assemblages we identified a minimum number of 80,221 individuals and 23 marine mollusc species (Supplementary Table 3). The limpets *Patella* spp. and the topshells *P. lineatus* together represent ca. 95% of the total Minimum Number of Individuals (MNI) for all stratigraphic units. The Simpson’s Diversity Index obtained for all units (ca. 0.7) confirms that only a few species dominate the taxonomic assemblage. The results also showed changes in the abundance of the warm-adapted topshell *P. lineatus* and the cold-adapted limpet *P. vulgata* over time (Fig. 4a), with four major shifts found throughout the shell midden sequence. The most represented species during the formation of the oldest unit of the shell midden (ca. 8825 cal BP) was the limpet *P. vulgata*, while the topshell *P. lineatus* was the most exploited species during the middle part of the sequence (ca. 8785–8300 cal BP). Subsequently, the number of *P. lineatus* topshells showed a notable decrease, while the limpet *P. vulgata* increased, becoming once again the most exploited species (ca. 8255–8020 cal BP). There was a drastic decrease in *P. vulgata* for the most recent units (ca. 8005–7655 ka cal BP), while *P. lineatus* and the limpet *P. depressa* increased in number, the latter becoming the most exploited limpet.

### Shell size variations

The dimensions of a total of 20,244 *P. lineatus*, 11,032 *P. vulgata* and 4549 *P. depressa* specimens were measured (Supplementary Table 4). The biometric results showed significant diachronic variations in the average shell size per unit for both limpets and topshells (Fig. 4b; Supplementary Fig. 3). The profiles of the three main species reported similar patterns in shell size variations over time. A clear and abrupt decrease in shell size was observed starting at ca. 8300 cal BP, reaching minimum sizes between ca. 8100 and 8070 cal BP. Following this, sizes began to increase again, reaching similar values to those observed prior to the 8.2 ka event between ca. 7935 and 7870 cal BP. An additional abrupt decrease in shell size was observed for the three taxa between the previous period and ca. 7655 cal BP.

## Discussion

The abrupt 8.2 ka cold event has been widely described from Greenland and North Atlantic records^5,6,8–11^. However, its impact in shelf coastal areas is scarcely documented, and current chronological resolution of the majority of marine records is insufficient to precisely determine the timing of this event. Bayesian modelling of a total of 65 radiocarbon dates from El Mazo allowed us to achieve the highest-chronological resolution for a Holocene sequence in an archaeological shell-midden located in the North Atlantic (Fig. 2; Supplementary Fig. 2; Supplementary Data 1), providing a unique opportunity for understanding the impact of this abrupt climate change on a regional marine environment, mollusc species diversity and composition, human behaviour, and the resilience of some of the last littoral foragers in this part of the world.

The 8.2 ka event has been dated in the NGRIP ice core to between 8250 and 8090 cal BP^6^ (Fig. 4f). However, recently, two North Atlantic SST cooling pulses have been identified within the event, as a result of a slowed AMOC^40^ during two outbursts of the proglacial Lakes Agassiz and Ojibway. These outbursts have been observed in marine records (at 8320 and 8202 cal BP, respectively)^7,41^ and Iberian speleothem isotopic records (at 8350–8340 and 8221–8211 cal BP, respectively)^42^. The stratigraphic sequence at El Mazo covers the interval between 9 and 7.4 ka cal BP and, according to the Bayesian model, includes several units formed during the 8.2 ka event. Among them, units 105 (8315–8185 cal BP) and 112C (8180–8070 cal BP) were selected for reconstructing SST in the northern Iberian coast and determining whether the 8.2 ka event had an impact in the region. The results from δ^18^O_shell_ values (Figs. 3 and 4d), clearly demonstrate that colder SST existed during the 8.2 ka event. Beyond an average annual SST cooling of ‒1.8 °C with respect to the previous unit, sequential isotopic analysis revealed the seasonal impact of the 8.2 ka event on coastal SST for the first time. The decline in littoral SST is, mostly, observable in the summer season (‒2.3 °C), with a smaller winter decrease (‒0.6 °C) across a period of two centuries (Fig. 3). These differential impacts are likely linked to differential seasonal insolation variations during the Early Holocene (Supplementary Text 3; Supplementary Fig. 4)^43^.

The analysis of mollusc species representation throughout the archaeological sequence at El Mazo has provided significant information on the impact of decreasing SST on mollusc populations during the 8.2 ka event. Results show that the warm-adapted topshell *P. lineatus* considerably decreased in abundance between ca. 8255 and 8020 cal BP, while the cold-adapted *P. vulgata* increased, becoming the most exploited species during this time (Fig. 4a). Warm-adapted species did not recover a similar percentage to the pre-8.2 ka event period until ca. 7935 cal BP (Fig. 4a). Therefore, climatic change had an important impact, not only on the mollusc population numbers of each species, but also on human foraging behaviour, by conditioning the food supplies available for consumption by Mesolithic societies. In contrast to *P. lineatus*, the warm-adapted limpet *P. depressa* showed a gradual increase over time, including during the 8.2 ka event (Fig. 4a). This is a consequence of the species-specific SST tolerance. While *P. depressa* is a warm-adapted species, this limpet has a higher resistance to colder temperatures than the topshell *P. lineatus*^23^, explaining why *P. depressa* continued to thrive during the 8.2 ka event.

A decrease in shell size was also observed at El Mazo during this colder period, something that has been variously linked to either changes in SST^37^ or a higher human pressure on coastal resources^44^, mostly in the form of demographic increase and additional resource pressure^45^. At El Mazo, a clear reduction in shell size for the three most dominant mollusc species can be seen during the 8.2 ka event. Given that each of these species have well-documented differences in temperature tolerance^23,26^, it seems that changes in SST cannot be considered responsible for changes in shell size. The biometric profiles obtained from the three species show a significant correlation between them, particularly between *P. depressa* and *P. vulgata* (R^2^ = 0.75; *p* < 0.0001). In addition, changes in shell sizes induced by variations in shore morphology and sea level rise over time are not expected, since this was very similar throughout the study period (Supplementary Text 4). Regarding the impact of the 8.2 ka event on the abundance of molluscs, it has been suggested that changes in upwelling, nutrients, and marine productivity could influence the abundance of marine resources available on the coast^20^. However, a recent study has suggested that ΔR values, which can be used as a proxy for upwelling, were not significantly different during the formation of stratigraphic units dated around the 8.2 ka event compared to the ΔR value derived from a unit dated at ca. 8.4 ka^28^. As a result, the reduction of shell sizes in littoral areas at 8.2 ka does not seem to be linked to natural marine processes. It therefore appears that, while SST influenced the abundance of different species at the site, the main cause for the decrease in their size was related to changing anthropogenic pressures.

Diachronic estimates of SST obtained from δ^18^O_shell_ measurements (Figs. 3 and 4d) and from the distribution of the main mollusc species (Fig. 4a) reveal, for the first time, colder surficial water masses between ca. 8255 and ca. 7935 cal BP in northern Iberia. We interpret this as a local response to the 8.2 ka event, consistent with the cooling recorded by global proxies (e.g., δ^18^O from Greenland ice cores)^5,6^. Based on the current chronological information (Fig. 2), SST decrease in northern Iberia probably began before that observed in the Greenland ice cores (8250 cal BP). Warm-adapted and cold-adapted species abruptly decrease and increase, respectively, from ca. 8255 cal BP, although this changing trend probably started earlier (ca. 8355 cal BP) (Fig. 4a; Supplementary Text 5). Pollen records from El Mazo also show the same trend^38^. Tree pollen decrease also started ca. 8355 cal BP, with more substantial decreases occurring after 8250 cal BP (Fig. 4e; Supplementary Text 5). This suggests that the two SST cooling pulses, which occurred in the North Atlantic as a consequence of the drainage of the proglacial Lakes Agassiz and Ojibway^7,41^, also had an impact on Iberian coastal locations, in agreement with speleothem isotopic records for this area^42^ (Supplementary Text 5). On the other hand, the impact of the 8.2 ka event on SST also seems to end later than previously observed in Greenland records (8090 cal BP). Both warm- and cold-adapted mollusc species do not recover to their previous abundance until between ca. 7975 and 7935 cal BP (Supplementary Text 5). Considering the usually rapid responses of these taxa to climate change^23–25^, the local effect of the 8.2 event on oceanographic conditions seems to have continued until after 8 ka cal BP in northern Iberia. This chronological timescale agrees with multiple proxies from Atlantic Europe, which reported a longer impact of this climatic anomaly than has been observed in Greenland^5^. It also appears that coastal environments recovered faster than interior settings following the 8.2 ka event. Studies previously conducted on pollen^38,46,47^ and speleothems^48,49^ from northern Iberia suggest that terrestrial ecosystems only started to return to pre-8.2 ka states between 7780 cal BP and 7655 cal BP (Fig. 4e, Supplementary Text 5).

Reconstruction of SST from δ^18^O_shell_ values revealed that the seawater temperature decreased ca. –2 °C during the 8.2 ka event in northern Iberia. A similar SST decrease has been recorded based on changes in the abundance of planktonic foraminifera species in the southern Landes Plateau (Bay of Biscay)^8^ and in North Atlantic marine locations^50^. Mathematical models have also predicted the SST cooling of coastal waters in northern Iberia^41,51^, suggesting connections between northern Iberia and the Nordic oceanic realm as part of a common driving mechanism linked to the North Atlantic Current^8^. The SST variations estimated from δ^18^O_shell_ values reported in this study follow a similar pattern to that seen for the vegetation observed in the El Mazo pollen records^38^, which showed a decrease in the percentage of arboreal pollen during the 8.2 ka event (Fig. 4e). Meanwhile, the proportion of pine forest during this period, favoured by colder climate conditions, and other meso-mediterranean species, also increased as a result of more arid and colder conditions^38^. Our marine proxy results also agree with additional palaeoclimatic information previously published for northern Iberia from terrestrial proxies^46–49^. Research previously conducted on pollen remains^46,47^ and speleothems^48,49^ recovered from sites located along the N Iberia has also revealed more arid and colder conditions during this period, suggesting that the 8.2 ka event had impacts for both marine and terrestrial environments exploited by humans in this Atlantic littoral area.

Previous studies in western Europe have hotly debated the impact of the 8.2 ka event, and its associated environmental changes, on human behaviour^16,52^. On the Iberian Peninsula, Bicho et al.^20^ reported changes in subsistence strategies, human settlement and technology in the coastal areas of central Portugal after the 8.2 ka event. In addition, research conducted in the Ebro Valley basin showed that, although not all of the valley was depopulated, several settlements were abandoned in the central part of the valley due to colder and drier climate conditions during the 8.2 ka event. This abandonment has been associated with a migration of Mesolithic populations towards less climate sensitive areas located in other parts of the Ebro Valley^53^ and/or along the Mediterranean coast^54,55^. In northern Iberia, archaeological sites located in the mountainous inland areas seem to have been occupied with lower intensity during the harsher period of the 8.2 ka event. The site of El Espertín does not report robust evidence of occupation from 8.4 to 8.0^56^. Only after 8.0 ka cal BP does a clear signal of human occupation emerge in the mountain areas located to the south of the Cantabrian region^56,57^. Given the higher levels of human predation on littoral resources observed during this time at El Mazo, it seems highly likely that the northern Iberian coast, a less climatically sensitive region than the nearby mountain areas^46^ and Ebro river basin^54,55^, provided a refuge for humans moving from inland regions. A similar scenario has also been argued for this region during the Last Glacial Maximum^58^ when northern Iberia became a refugium for forager groups. Population migrations from adjacent areas are also in agreement with the growing presence of geometric microliths seen in northern Iberia from ca. 8.0–8.2 ka cal BP^59,60^. Increasing human pressure on coastal environments during the 8.2 ka event is suggested by decreasing mollusc size, as well as evidence for the growing exploitation of lower and more exposed areas during this period (Fig. 4c), which are continually wave-beaten, being therefore more dangerous than higher intertidal zones^44^. Furthermore, recent palaeo-demographic studies based on summed probability distributions of calibrated radiocarbon dates have recorded a demographic increase in northern Iberia and Atlantic Portugal from 8.2 ka ago, coincident with a population decrease in the Ebro valley^61^. This process has been related to an increased use of marine resources in Portugal^62^. Thus, it seems that coastal sites, like El Mazo, provided more stable, reliable, and predictable environments for human foragers in Atlantic Iberia, with populations moving towards the coast from the increasingly unpredictable interior.

The benefits of increased human use of coastal areas during this climatic event are clear. However, importantly, growing human pressures, and populations, along the northern Iberian coast during this part of the Early Holocene do not seem to have led to obvious overexploitation of coastal resources, despite evidence for an overall decline in mollusc size. Larger specimens are continuously and systematically harvested, instead of smaller taxa, throughout the whole sequence of occupation (Supplementary Text 6, Supplementary Table 5). In addition, limpet sizes never decrease, at least at the overall population level, to 20 mm or lower (Supplementary Fig. 3), the minimum size currently established by the regulations in order to guarantee the survival of these species over the long-term in the area (Boletín Oficial de Cantabria, BOC-2017-4672, https://boc.cantabria.es). Similarly, scheduled littoral exploitation patterns year-round, which were conceived to maximize the meat yield extracted from molluscs, continued to be practiced despite greater intensification in the use of available resources during the 8.2 ka event^22^. Our results therefore suggest an ongoing application of local marine ecological knowledge by some of the last foragers in western Europe, despite major climatic and demographic changes, with foragers continuing to apply sustainable resource management strategies.

This study highlights that taxonomic, sclerochronological, geochemical and chronological analyses of molluscs from archaeological sites have the potential to uncover direct insights into the impacts of climatic changes on human-relevant local marine environments. Not only that, but the high seasonal resolution of the climate proxies employed enables the seasonal impacts of the 8.2 ka event to be directly observed. The detailed chronology of El Mazo, as well as these high-resolution proxies, provide a more refined linkage between changing environmental conditions and human behaviour in this region during the 8.2 ka event. Based on comparison with inland records derived from palynological studies and geochemical analyses of speleothems, it seems that coastal regions provided crucial refugia for growing human populations bolstered by migrations from the interior. The resolution provided by this methodology has major implications for other studies seeking to determine the significance of climate change for marine environments, and human adaptations, in regions, and periods of time, where ocean resources were crucial to our species and its hominin relaltives^44,63,64^. Furthermore, understanding the implications for marine ecology and human behaviour of colder and drier conditions provoked by climatic events such as that seen 8.2 ka ago in North Atlantic Europe^5,9,10^, can provide detailed clues to the magnitude and nature of future climate changes, lags in their manifestation between different regions and environments, and their ultimate outcomes for human societies. This is especially the case given that the AMOC is currently weakening^65^. As the palaeosciences become increasingly recognised by earth scientists, policy makers and climate prediction scientists as an important, detailed source of data for climate predictions beyond anecdotal comparisons, high-resolution methodologies, like that presented here, will become increasingly important.

## Methods

### Radiocarbon dating and Bayesian modelling

An outlier Bayesian chronological model for El Mazo was implemented using the OxCal v.4.4.2 software^33,34^. Radiocarbon calibrations relied on the IntCal20 calibration curve for the northern hemisphere^35^ and on the marine calibration curve from Heaton et al.^36^. Corrections for marine radiocarbon reservoir effects (ΔR) in shell samples from northern Iberia followed the recommendations in García-Escárzaga et al.^28^. According to that study, a different ΔR value should be used depending on the mollusc species: –114 ± 170 for *P. vulgata* and –257 ± 120 for *P. lineatus*. A stratigraphic model was defined by grouping samples from the same archaeological layer within a phase with start and end boundaries in accordance with OxCal terminology. These were set in a sequence following the available stratigraphic information. The OxCal model code is given in Supplementary Code 1. The Bayesian model was run 4–5 times and results compared to assess reproducibility, which was observed to be present for scales of ca. 5–10 years and consequently all reported dates here were rounded to the nearest lustrum. The obtained chronological sequence was compared with the Greenland Oxygen Isotopic record (NGRIP)^6,39^. We used the OxCal Date command to provide a reference for an undated object within a phase. This is also used to provide a rough estimate of the chronology of each phase in this study. Estimates of the start, end, and duration of each phase, plus dates for each object within the stratigraphic units, are given in Supplementary Data 1.

### Stable oxygen isotope analysis on *Phorcus lineatus*

A total of 27 shells from the topshell *P. lineatus*, which were recovered from five different stratigraphic units (108, 107, 105, 112C and 101B) and located at different points within the shell midden sequence of El Mazo (Fig. 1d), were subjected to stable oxygen isotope (δ^18^O) measurements to reconstruct sea surface temperature (SST) diachronic variations. Four specimens were analysed per stratigraphic unit, except for SUs 108 and 112, where ten and five shells were selected, respectively. According to modern baseline isotopic studies previously published for this species in northern Iberia^31,32^, specimens smaller than 17 mm in diameter were selected to avoid growth stoppages, which would preclude the reconstruction of annual minima and/or maxima temperatures. All shells were measured using a digital calliper to the nearest 0.01 mm. Prior to sampling the shell samples for oxygen isotope analysis, the samples were first placed in 30 vol% H_2_O_2_ for 48 h to remove organic matter from the shells following a frequently-applied methodology^22,31,32^. The cleaned shells were then air-dried at ambient temperature.

The methods applied to extract micro-samples of calcium carbonate from the aragonite layers of shells for δ^18^O analyses were similar to those previously employed for this species^22,31,32^. A first sample was taken from inside the edge of the shell aperture (Supplementary Fig. 5) to avoid breaking the edge when removing the outer layers (i.e. *periostracum* and calcite layer). The outer layers were removed using a Dremmel microdrill with a 2 mm drill bit. The remaining samples were taken sequentially along the whorl, but from the outside of the shell (Supplementary Fig. 5). All calcium carbonate micro-samples were taken manually from the aragonite layer using a dentist microdrill with a 0.5 mm drill bit coupled to a stereoscopic microscope. The sampling spots were separated by 0.3 mm, and the carbonate recovered weighed approximately 150–200 μg. A total of 45 calcium carbonate micro-samples were taken from each shell. The only exception made was for the shells recovered from SU 108, where a total of 30 samples were taken from the shell edge. This give a total of 1065 calcium carbonate micro-samples taken for oxygen stable isotope analyses.

Micro-samples taken on the ten shells recovered from SU 108 (n = 300) and on two shells from the other four stratigraphic unit (MA.107.1, MA.107.2, MA.105.1, MA.105.2, MA.112.1, MA.112.2, MA.101.1, MA.101.2) (n = 360) were analysed using an IRMS Thermo Scientific MAT 253 coupled to Kiel device at the University of Michigan (USA). The instrumental error was better than ± 0.12‰. Micro-samples taken on the rest of the nine shells analysed herein (MA.107.3, MA.107.4, MA.105.3, MA.105.4, MA.112.3, MA.112.4, MA.112.5, MA.101.3, MA.101.4) (n = 405) were analysed using an IRMS Thermo Scientific MAT 253 coupled to Kiel IV device at the Complutense University of Madrid (Spain). In this case, the instrumental error was better than ± 0.03‰. In both cases, the powder samples were dissolved with concentrated phosphoric acid at 70 °C and isotopic ratios were calibrated against NBS-18 (‒23.2‰) and NBS-19 international standards (‒2.20‰). The results are reported as δ^18^O (‰) relative to the Vienna Pee Dee Belemnite (VPDB) reference standard.

### Calculation of past sea surface temperatures (SST) from δ^18^O_shell_ values

Following previous investigations conducted on topshell *P. lineatus* species in northern Iberia^31,32^, the water-aragonite fractionation factor obtained by Kim et al.^66^ for synthetic aragonite was used to estimate the SST during the mollusc life span:

1 $${1}000{\text{ln}}\alpha \, = { 17}.{88 }* \, \left( {{1}0^{{3}} /{\text{ T}}} \right) \, - { 31}.{14}$$

where T corresponds to SST in Kelvin and α is the fractionation between water and aragonite described by the equation:

2 $$\alpha \, = { 1}000 \, + \, \delta^{{{18}}} {\text{O}}_{{{\text{shell}}}} \left( {{\text{SMOW }}\textperthousand} \right) \, /{ 1}000 \, + \, \delta^{{{18}}} {\text{O}}_{{{\text{water}}}} \left( {{\text{SMOW }}\textperthousand} \right)$$

To estimate the δ^18^O_water_ values, we employed the correction proposed by Fairbanks^67^. Fairbanks^67^ proposed a gradual correction of + 0.011‰ for each metre of sea level rise. In this investigation, the current annual δ^18^O_water_ value average (0.90‰)^31,32^ was used to estimate the δ^18^O_water_ value for the past. To calculate the sea level height during the formation of each stratigraphic unit, radiocarbon dates obtained for each stratigraphic unit and previously published sea level rise estimates for northern Iberia through Early Holocene^68^ were employed. Nevertheless, another additional correction should be applied in order to accurately estimate δ^18^O_water_ values during the past, as the correction developed by Fairbanks did not consider the possibility of short-term changes, including the ‘8.2 ka event’. Warmer conditions from the Early Holocene led to the collapse of the Laurentide ice saddle and the Agassiz-Ojibway palaeolakes, and the posterior drainage of cold freshwater, with a very low δ^18^O signal, into the North Atlantic^7^. Indeed, the 8.2 ka event was caused by a depletion of the thermohaline circulation in the North Atlantic derived from this freshwater mass released into the ocean. This process, radiocarbon dated to around 8.33 cal BP^7^, led to a depletion of δ^18^O_water_ values in the North Atlantic, with a reduction of between –0.2 and –0.4‰ estimated for northern Iberia^41,69^. Research conducted in our study area applying δ^18^O analyses on speleothems showed negative shifts in δ^18^O_precipitation_ around this time, which has been explained as a local signal of the lower δ^18^O of sea surface waters^48^. Therefore, in this study, δ^18^O_water_ values for those units affected by the meltwater discharge were estimated by subtracting 0.3‰ from the values estimated using the Fairbanks correction^65^.

### Archaeomalacological analyses

The archaeological shells studied here were recovered from the shell midden site of El Mazo cave (N Spain), from 23 stratigraphic units located in the excavation squares X15 and X16, covering the entire chronological sequence. Archaeological materials were recovered across a surface area of 0.5 m^2^. The total volume of sediment excavated and analysed between all stratigraphic units was 293.5 L and the remains employed in our study were recovered using mesh screen sizes of 1, 2, and 4 mm. Anatomic and taxonomic identification and quantification were performed in order to establish species distribution and their relative abundance in the different stratigraphic units. A total of 262,547 mollusc remains were classified into different fragmentation categories following the methodology proposed by Gutiérrez-Zugasti^70,71^. The fragmentation categories employed are different for bivalves, spiralled gastropods and non-spiralled gastropods. For bivalves: valves (complete, COMV; fragmented valve, FRAV; fragment with complete hinge, FCG; and anterior/posterior hinge fragments, AHF/PHF; separated into right and left) and fragments. For spiralled gastropods: complete individual (COMI), fragmented individual (FRAI), apical fragment (API), stoma fragment (STOF), umbilical fragment (UMBF) and fragments (FTS). For non-spiralled gastropods: complete individual (COMI), fragmented individual (FRAI), apical fragment (APIF) and fragments (FTS). This procedure, based on non-repetitive elements, is similar to those proposed by other scholars for other taxa in other littoral areas^72^. The minimum number of individuals (MNI) for each taxon was calculated using the corresponding formulae: (i) Bivalves: COMV + FRAV + FCH + (AHF or PHF, whichever is greater). First, the right and left valves are counted separately, and the MNI is the greater of the two totals. (ii) Spiralled gastropods: COMI + FRAI + [APIF or (STOF + UMBF), whichever is greater]. (iii) Non-spiralled gastropods: COMI + FRAI + APIF. The species nomenclature applied in this study was that proposed by the World Register of Marine Species (WoRMS, http://www.marinespecies.org/). Taxonomic identification was performed using different reference manuals and the comparative collection of the Institute of Prehistory of the University of Cantabria (IIIPC). Limpet remains that were not assigned to any *Patella* species as a consequence of taphonomic processes, which precluded us correctly classifying every APIF and FTS, were denoted as *Patella* sp. Finally, in order to accurately estimate the number of each limpet species and to avoid the artificial effects caused by taphonomic processes in the estimation of *Patella* taxa, the MNI corresponding to *Patella* sp. was assigned to each limpet species according to its percentage representation in each unit.

A biometric analysis was also performed (i) to identify changes in shell size through time, (ii) to determine the collection areas of the limpet *P. vulgata*, and (iii) to establish whether there was any kind of shell size selection during collection. Measurements were taken using digital callipers to the nearest 0.01 mm and followed the protocols established for gastropods^71^. The collection areas were defined using the equations proposed by Bailey and Craighead^37^ for *P. vulgata*. The degree of exposure of the coast where the shells were collected was calculated using the following equation: Length (0.142) – Height (0.06) + Width (0.0489) – 5.328. When the results were lower than –0.15, the individuals were assigned to exposed areas, while results higher than that limit were assigned to sheltered shores. To determine the intertidal zone where *P. vulgata* specimens were collected, length/height (L/H) ratios were calculated from each limpet. Then the control value obtained by Gutiérrez–Zugasti^71^ for northern Iberia was applied to determine whether the specimens were harvested in the lower or higher intertidal zone. In order to establish the occurrence of size selection during collection, a normality test (Shapiro–Wilk) was applied under the assumption that non-normal distributions reflect some kind of shell size selection^71^. The statistical tests were performed using the software PAST. Stratigraphic units where the number of measurable specimens was lower than 30 were not considered for the biometric study.

## Supplementary Information

Supplementary Information 1.

Supplementary Information 2.

Supplementary Information 3.

## Data Availability

All of the data reported in this article is provided in the Supplementary Information, Tables, Figures, and Data files. Bayesian modelled results were included in Fig. 2, as well as in Supplementary Fig. 2 and Supplementary Data 1. All stable oxygen isotope values obtained from *Phorcus lineatus* (da Costa, 1778) mollusc species, as well as the sea surface temperatures (SST) estimated from these isotopic values, are provided in Supplementary Data 2. Mollusc species representation and shell sizes over time are included in Supplementary Table 3 and Supplementary Fig. 3, respectively. All other data supporting the findings and interpretations of this study are available in existing publications and the Supplementary Information provided alongside this manuscript. The archaeological remains studied and sampled for oxygen isotope analysis are curated in the Archaeology collection at the University of Cantabria (Santander, Spain). These materials are to be returned to Archaeological Museum of Asturias (Oviedo, Spain).
