# Supplementary material for: Human forager response to abrupt climate change at 8.2 ka on the Atlantic coast of Europe
Source: Sci Rep. 2022 May 2;12:6481. doi: 10.1038/s41598-022-10135-w (PMC9021199; doi:10.1038/s41598-022-10135-w)
Supplement: Supplementary file 1 — Supplementary Information 1. [file 41598_2022_10135_MOESM1_ESM.pdf]

Supplementary Information for

Human forager response to abrupt climate change at 8.2 ka on the Atlantic coast of Europe

Asier García-Escárzaga[1,2,3*], Igor Gutiérrez-Zugasti[4*], Ana B. Marín-Arroyo[5,6], Ricardo Fernandes[7,8], Sara Núñez de la Fuente[4], David Cuenca-Solana[4,9], Eneko Iriarte[10], Carlos Simões[11], Javier Martín-Chivelet[12], Manuel R. González-Morales[4] and Patrick Roberts[1,13].

[1] Department of Archaeology, Max Planck Institute for the Science of Human History. Jena, Germany.
[2] Departamento de Ciencias Humanas, Universidad de La Rioja. Logroño, Spain.
[3] Department of Prehistory and Institute of Environmental Science and Technology (ICTA), Universitat Autònoma de Barcelona. Bellaterra, Spain.
[4] Instituto Internacional de Investigaciones Prehistóricas de Cantabria, Universidad de Cantabria, Santander, Gobierno de Cantabria. Santander, Spain.
[5] Grupo de I+D+i EVOADAPTA, Departamento de Ciencias Históricas, Universidad de Cantabria. Santander, Spain.
[6] Department of Archaeology, University of Cambridge, Downing Street, Cambridge, CB2 3DZ, UK
[7] School of Archaeology, University of Oxford. Oxford, United Kingdom
[8] Faculty of Arts, Masaryk University. Brno, Czech Republic.
[9] Centre de Recherche en Archéologie, Archeosciences, Histoire (CReAAH), UMR-6566, Université de Rennes 1. Rennes, France.
[10] Laboratorio de Evolución Humana, Departamento de Historia, Geografía y Comunicación, Universidad de Burgos. Burgos, Spain
[11] ICArEHB - Interdisciplinary Center for Archaeology and the Evolution of Human Behaviour, Universidade do Algarve. Faro, Portugal.
[12] Facultad de Ciencias Geológicas, Universidad Complutense de Madrid & Instituto de Geociencias (CSIC-UCM). Madrid, Spain.
[13] School of Social Sciences, University of Queensland. Queensland, Australia.

* Corresponding author: garcia-escarzaga@shh.mpg.de; fernandoigor.gutierrez@unican.es

This PDF file includes:

Supplementary Text 1: The shell midden site of El Mazo (Asturias, N Spain)
Supplementary Text 2: Shell midden formation and micromorphology results
Supplementary Text 3: Seasonal impact of the 8.2 ka event in coastal SST
Supplementary Text 4: Coastal morphology in northern Iberia through the Mesolithic

Supplementary Text 5: Chronology of the 8.2 ka event in northern Iberia

Supplementary Text 6: Shell-size distributions.

Supplementary Figures 1 to 6

Supplementary Tables 1 to 6

Supplementary Code 1

Supplementary References

Supplementary Text 1. The shell midden site of El Mazo (Asturias, N Spain)

El Mazo is located in the town of Andrín, in the municipality of Llanes, in Asturias, Northern Spain (43° 24′ 4″ N, 4° 42′ 42″ W) (Fig. 1a). The current distance from the site to the coastline is around 1 km, although during the Mesolithic this distance would have varied due to sea level rise. Nevertheless, according to a previous study for the Cantabrian region[1,2], this distance was no larger than three kilometres during the last 9,000 years. The rockshelter is approximately 18 metres long and seven metres deep (Fig. 1b). Excavations were undertaken in the inner part of the rockshelter, close to the walls, in 2009, 2010 and 2012 (Fig. 1c). A 2 x 1 m test pit was opened in 2009–2010 in squares V15 and V16 and the resulting north profile was sampled in 2012 in a limited extension (2 m in the east-west axis x 0.25 m in the north-south axis), recording a total of 25 stratigraphic units in squares X15 and X16 (Fig. 1d). The excavation of the rockshelter area produced several stratigraphic units (SUs) composed mainly of shell midden deposits: SUs 100 to 108 and 110 to 122, except SUs 104, 112A, 117 and 122, which correspond to combustion structures (hearths) with high densities of charcoal, ash and burnt shells, suggesting that these fire structures were related to shellfish processing. Individual shell midden units were defined by their distinct sediment type and the number of shellfish remains. Sometimes these midden units were composed solely of crust, as a consequence of calcium carbonate precipitation over the shell midden, as was the case for SU 102 and SU 106 (see previous studies[2,3,4,5,6] for more information; a very detailed explanation of the stratigraphy can also be found in two previous investigations[3,6]).

Supplementary Text 2. Shell midden formation and micromorphology results

The Bayesian model developed herein from 65 radiocarbon dates obtained throughout the sequence of the shell midden site of El Mazo has reported a total of sixteen outliers (Supplementary Data 1). The radiocarbon dates obtained from these remains were not in

agreement (< 60%) with the modelled date for their respective stratigraphic unit. Three out of thirteen shells used for reconstructing SST and radiocarbon dating (two from unit 101B and one from unit 105) were considered as outliers by the Bayesian model. Consequently, these shells were not used for palaeoclimate discussion. This result might relate to the complex formation processes of shell middens, as well as postdepositional factors. Micromorphological analysis was conducted at El Mazo in order to clarify the origin of the stratigraphic layers, the nature of the complex stratigraphic contacts, and overall reconstruction of the depositional history of the shell midden[7].

Overall, the shell midden was accumulated through a successive alternation between anthropogenic accumulations and natural reworking of anthropogenic debris previously deposited elsewhere. Unit 101B revealed a superposition of different events of anthropogenic accumulations identified microstratigraphically (Supplementary Fig. 6). The more conspicuous sedimentary accumulations within 101B consist of a mixing of diverse anthropogenic debris (shells, charcoals, pebbles), suggesting intentional dumping of items derived from combustion, shellfish, and organic matter processing activities. The existence of superimposed, different accumulation events within the field unit 101B makes the context of the analysed shells reliable, allowing us to exclude major post-depositional physical disturbances. However, such anthropogenic dumping events may have resulted in the inclusion of some shells from different occupation, perhaps explaining the outlier detected by the Bayesian Model conducted in this investigation (Supplementary Data 1).

Unit 105, in turn, is an accumulation of anthropogenic debris by natural, colluvial-like processes that affected the site. The overall geometry of 105 suggests that a major erosional event affected all of the previous deposits, creating a steep slope towards the interior of the rockshelter. Several micromorphological samples were collected from the upper and lower contacts and from the middle of 105. These samples revealed that successive slope deposits, with variable sedimentary characteristics, accumulated over the sloping surface (Supplementary Fig. 6), with none of them apparently having a direct anthropogenic origin but rather being the result of natural reworking of occupations that might have occurred in other adjacent areas of the rockshelter away from this slope. These processes might also entail mixing of remains from different occupations in unit 105.

Overall, the micromorphological analysis shows that, within field units 101B and 105, different occupation events are represented, and that these deposits result from the mixing of different items by either anthropogenic or natural processes. These observations explain the existence of the dates which fall outside of the age modelled by the Bayesian statistical analysis, although deposits with lower reworking do occur in Mesolithic shell middens and can be traced micromorphologically[8,9] as seems to be the case of the modelled dates on 101B. Targeting such deposits through microstratigraphic control of isotopically analysed samples is essential in future work.

Supplementary Text 3. Seasonal impact of the 8.2 ka event in coastal SST

Due to the high temporal resolution of the climate proxy employed, the results were used to infer the seasonal impact of the 8.2 ka event on regional coastal SST for the first time. The decline in littoral SST is mostly observable in the summer season (–2.3 ºC), and notably less in winter (–0.6 ºC), across a span of two centuries (Fig. 3). This homogenous result through time contrasts with the available data derived from continental proxies in the North Atlantic area. Previous investigations have suggested different duration of the 8.2 ka event depending on the season, with colder conditions lasting longer in summers (ca. 160 yrs) than in winters (ca. 70 yrs)[10,11,12].

However, results obtained herein from a high-resolution palaeothermometer reveal, for the first time, that this climate anomaly affected every season during the duration of this cold event, but with a different cooling intensity. Taking into account that current seasonal SST variations along the northern Iberian coast are mainly controlled by seasonal insolation changes[13,14], the different seasonal SST signal between summer and winter during the 8.2 ka event was probably caused by seasonal insolation variations occurring during the Early Holocene (Supplementary Fig. 4)[15,16,17,18]. Decreasing summer insolation in the northern hemisphere[15,16] would produce colder summer SST that amplified the SST cooling due to North Atlantic water mass cooling, significantly lowering the recorded SST, as observed in reconstructed temperatures from *P. lineatus* $\delta^{18}O_{shell}$ data. By contrast, the higher winter SST, due to increasing winter insolation during the Early Holocene, would counteract (negative feedback) the SST cooling due to the North Atlantic cold-water mass, making SST cooling less evident in winter. Additionally, the effect of changes in oceanic scale currents (Atlantic meridional overturning circulation) or slope Navidad current (Iberian Poleward Current) could have also contributed to the observed

seasonal SST variability – the latter being responsible for the occasional intrusion of relatively warm surficial waters along the northern Iberian coast in winter, contributing to warmer winter SSTs[19,20,21,22].

Supplementary Text 4. Coastal morphology in northern Iberia through the Mesolithic

Mollusc species representation throughout the El Mazo shell midden sequence showed that *Phorcus lineatus* and the *Patella* genus together represent ca. 90% of MNI in all units. In addition, the presence of taxa collected from estuarine areas, such as clams, was very limited[6] (Supplementary Table 3). According to the ecological preferences of the different species recovered from the shell midden site[23,24,25], the results indicated that the Holocene coastline in the study area was characterised by exposed shores and limited estuarine environments. The biometric dataset and the application of the equations proposed by Bailey and Craighead[26] demonstrated that between 95 and 100% of the *P. vulgata* specimens measured in every stratigraphic unit were collected from exposed shores (Supplementary Table 6). Data obtained from shell species distribution and shell size measurements reveals that the morphology of the coastline was predominately a rocky exposed shore. These conditions were very similar throughout the Mesolithic period and persist along today's littoral in central northern Spain[25,27,28,29,30,31,32].

On the other hand, the impact of the sea level rise in the foraging opportunities, the mollusc assemblage composition and the shell sizes should have been very minor. Bayesian modelling conducted in this study has revealed that the shell midden site of El Mazo cave was formed during a total period of 1,600 years, from 9,000 to 7,400 cal BP. The first complete Holocene sea-level curve obtained for northern Iberia has shown that sea level rose ca. 10 m during this period[1]. Due to the narrow coastal platform in northern Iberia, the distance from the site to the coastline was no larger than 2-3 kilometres and this did not significantly change during the 8.2 ka event.

Supplementary Text 5. Chronology of the 8.2 ka event in northern Iberia

The 8.2 ka event has been dated from 8,250 to 8,090 cal BP in the Greenland ice cores (Fig. 4f)[33,34]. However, differences in timescales have been found at lower latitudes. Rohling and Palike[11] reported lower intensity, but longer climate anomalies along the North Atlantic, for

example. Information available so far from northern Iberia also displays chronological differences compared to the Greenland dates[35,36,37,38,39]. In this study, SST estimated from $\delta^{18}O_{shell}$ placed the beginning of the colder conditions between the unit 107 (8,550–8,330 cal BP; ca. 8,435) and the unit 105 (8,315–8,185 cal BP; ca. 8,255 cal BP). The end of the climate anomaly, according to the SST measurements, occurred at some time between the units 112C (8,180–8,070 cal BP; ca. 8,125 cal BP) and 101B (7,945–7,795 cal BP; ca. 7,875 cal BP) (Fig. 3; Supplementary Fig. 2; Supplementary Data 1). Changes in the main species representation over time were crucial in supporting, in combination with the radiocarbon dating, the identification of the 8.2 event in the sequence (Fig. 4a).

The slight decrease of the warm-adapted *P. lineatus* and the increase of the cold-adapted *P. vulgata* during the formation of unit 111 (8,455–8,255 cal BP; ca. 8,355 cal BP), followed by an abrupt amplification of this trend in the chronological range of unit 105 (8,315–8,185 cal BP; ca. 8,255 cal BP), suggested that this climate anomaly could have begun in the study area before that observed from the Greenland record (8,250 cal BP)[33,34]. This is in agreement with the two North Atlantic SST cooling pulses previously described from marine records (8,320 and 8,202 for the two main outbursts)[40,41,42,43,44,45,46], but also from the northern Iberian speleothem isotopic records[35]. Two prominent falls within the stable oxygen isotope series published by Dominguez-Villar et al.[35] from an annual laminated stalagmite from northern Iberia (8,350-8,340 and 8,221-8,211 cal BP) represent the local response of the two SST cooling pulses occurred in the North Atlantic as a consequence of the drainage of proglacial lakes Agassiz and Ojibway. In contrast to studies previously referenced, Greenland ice cores did not record this first cooling pulse[33,34] despite of being a high-resolution subdecadal climate archive. Following to Dominguez-Villar[35], this discrepancy could be explained alluding to the increase in sea ice at high latitudes, which possibly counteracted the $\delta^{18}O$ freshwater anomaly in those regions during the first cooling pulse.

According to mollusc species distributions, SST in northern Spain probably started to increase during the formation of the unit 102 (8,150–8,045 cal BP; ca. 8,070), when *P. lineatus* and *P. vulgata* showed an increased and decreased trend, respectively (Fig. 4a). This suggests that the 8.2 ka event had its maximum effect (i.e., minimum SST) in northern Iberia slightly later than that observed in Greenland (8,180 cal BP)[34]. In spite of both *P. lineatus* and *P. vulgata* taxa representation starting to change from ca. 8,070 cal BP in northern Iberia, cold- and warm-adapted species did not recover to the percentages seen prior to the 8.2 ka event until units

101D (8,010–7,945 cal BP; ca. 7,975 cal BP) and 101.1A (7,985–7,885 cal BP; ca. 7,935 cal BP), respectively. Considering the fast response of these mollusc species to current climate changes[47,48,49,50] along the European Atlantic façade, and the delayed return of these species to pre-8.2 ka levels in the archaeological record, it is possible that the local effect of the 8.2 ka event on oceanographic conditions continued until after 8 ka cal BP in N Iberia.

From a terrestrial point of view, the patterns of vegetation observed at El Mazo from pollen analysis[51] show a prominent decrease in the percentage of arboreal pollen in several units, dated between ca. 8,255 cal BP and ca. 7,655 cal BP (Fig. 4e). Nevertheless, as was observed from the mollusc species representation analysis, a slight decrease of tree pollen occurred prior to the prominent decrease seen in unit 105 (8,315–8,185 cal BP; ca. 8,255 cal BP). Arboreal pollen began to decrease after the formation of unit 111 (8,455–8,255 cal BP; ca. 8,355 cal BP), supporting a possible effect of two SST cooling pulses in coastal areas of northern Iberia. In addition, the arboreal pollen did not recover to proportions similar to those observed prior to the 8.2 ka cold event until the most recent unit of the sequence (7,800–7,400 cal BP; ca. 7,655) (Fig. 4e), highlighting that the terrestrial ecosystem recovered later than the marine habitats. Likewise, rainfall variations observed from the Asiul speleothem[38] showed that the climate anomaly affected terrestrial areas until 7.7 ka cal BP. According to these data, arboreal mass did not recover until ca. 7.6 ka cal BP in northern Iberia, although tree pollen started to increase around ca. 7.9 ka cal BP[51,52] (Fig. 4e).

Supplementary Text 6. Shell size distributions

A Shapiro-Wilk normality test was conducted on the shell sizes of *Phorcus lineatus* and *Patella* spp. from different stratigraphic units. Results showed differences in size distributions between taxa and through time. A normal distribution represents a biological population, and it has been traditionally linked to mollusc exploitation without size selection. In contrast, a population not normally distributed would suggest a collection pattern based on a preference of specific sizes. Non-symmetrical distributions with positive skew (deviated towards the larger sizes) have been interpreted as evidence of size selection. Previous investigations have argued that a random size collection should be considered as evidence for a higher intensification in littoral resource exploitation patterns, while a selection of larger specimens could be a signal of conscious resource management by Mesolithic foragers aimed at not over-exploiting mollusc populations[6,25,53,54,55,56,57]. Results obtained herein from the shell size analysis of the three main

species recovered from El Mazo have suggested the existence of different mollusc collection strategies throughout the shell midden sequence (Supplementary Table 5). Nevertheless, the majority of the stratigraphic units showed shell size distributions that were not normally distributed, especially for *P. lineatus* and *P. vulgata* species. Only *P. depressa* displayed a consistent exploitation pattern throughout the first centuries of shell midden formation (ca. 8.9k - 8.2k cal BP) that was not based on a selection of larger shells (Supplementary Table 5). However, this pattern is probably not related to a higher exploitation of this species during this time. The exploitation of *P. depressa* species without size selection is not common in the more recent stratigraphic units, including within those which had reported a high pressure over littoral resources based on shell size reduction measurements (Fig. 4b). This collection pattern of *P. depressa* during the Early Holocene was probably caused by the low overall frequency of this species in the northern Iberian coast (Fig. 4a; Supplementary Table 3). Those colonies of individuals with a low population, as occurred with *P. depressa* before starting the Middle Holocene (Fig. 4a), avoid the existence of specimens unusually larger than the average (Supplementary Fig. 3), leading to rise to populations normally distributed.

Supplementary Figure 1. Stratigraphy of the inner test pit (square X15 and X16). Limits of each stratigraphic units have been marked with white lines.

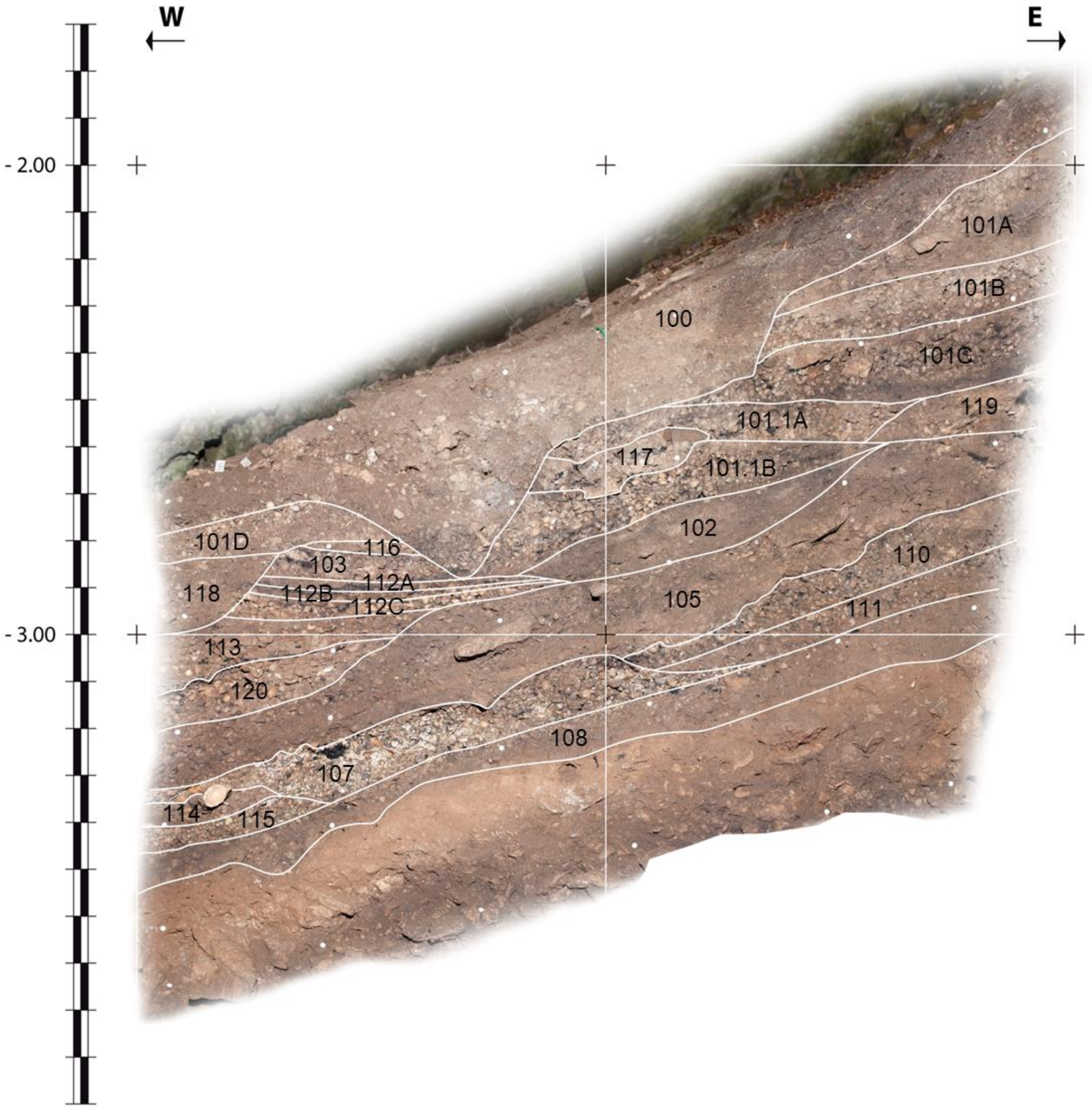

Supplementary Figure 2. Bayesian model of radiocarbon dates for the Mesolithic sequence at El Mazo. Dates on animal collagen and plant remains are shown in purple and marine shells are shown in green. This model has twenty-three separate phases corresponding to the stratigraphic sequence, from the bottom unit 108 to the top unit 100. The 8.2 ka event has been identified in several units at the site. OxCal CQL code is provided in the Supplementary Code 1.

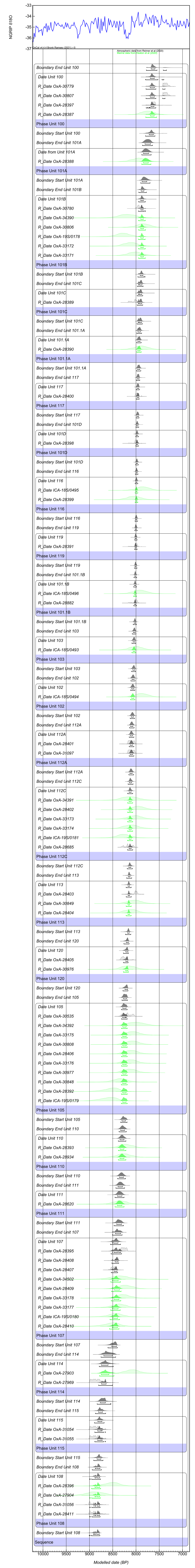
NGRIP δ18O
-33
-34
-35
-36
-37
OxCal v4.4.4 Bronk Ramsey (2021); r:5
Atmospheric data from Reimer et al (2020)
Marine data from Heaton et al (2020)
Boundary End Unit 100
Date Unit 100
R_Date OxA-30779
R_Date OxA-30807
R_Date OxA-28397
R_Date OxA-28387
Phase Unit 100
Boundary Start Unit 100
Boundary End Unit 101A
Date from Unit 101A
R_Date OxA-28388
Phase Unit 101A
Boundary Start Unit 101A
Boundary End Unit 101B
Date Unit 101B
R_Date OxA-30780
R_Date OxA-34390
R_Date OxA-30806
R_Date OxA-19S/0178
R_Date OxA-33172
R_Date OxA-33171
Phase Unit 101B
Boundary Start Unit 101B
Boundary End Unit 101C
Date Unit 101C
R_Date OxA-28389
Phase Unit 101C
Boundary Start Unit 101C
Boundary End Unit 101.1A
Date Unit 101.1A
R_Date OxA-28390
Phase Unit 101.1A
Boundary Start Unit 101.1A
Boundary End Unit 117
Date Unit 117
R_Date OxA-28400
Phase Unit 117
Boundary Start Unit 117
Boundary End Unit 101D
Date Unit 101D
R_Date OxA-28398
Phase Unit 101D
Boundary Start Unit 101D
Boundary End Unit 116
Date Unit 116
R_Date ICA-18S/0495
R_Date OxA-28399
Phase Unit 116
Boundary Start Unit 116
Boundary End Unit 119
Date Unit 119
R_Date OxA-28391
Phase Unit 119
Boundary Start Unit 119
Boundary End Unit 101.1B
Date Unit 101.1B
R_Date ICA-18S/0496
R_Date OxA-28882
Phase Unit 101.1B
Boundary Start Unit 101.1B
Boundary End Unit 103
Date Unit 103
R_Date ICA-18S/0493
Phase Unit 103
Boundary Start Unit 103
Boundary End Unit 102
Date Unit 102
R_Date ICA-18S/0494
Phase Unit 102
Boundary Start Unit 102
Boundary End Unit 112A
Date Unit 112A
R_Date OxA-28401
R_Date OxA-31097
Phase Unit 112A
Boundary Start Unit 112A
Boundary End Unit 112C
Date Unit 112C
R_Date OxA-34391
R_Date OxA-28402
R_Date OxA-33173
R_Date OxA-33174
R_Date ICA-19S/0181
R_Date OxA-28685
Phase Unit 112C
Boundary Start Unit 112C
Boundary End Unit 113
Date Unit 113
R_Date OxA-28403
R_Date OxA-30849
R_Date OxA-28404
Phase Unit 113
Boundary Start Unit 113
Boundary End Unit 120
Date Unit 120
R_Date OxA-28405
R_Date OxA-30976
Phase Unit 120
Boundary Start Unit 120
Boundary End Unit 105
Date Unit 105
R_Date OxA-30535
R_Date OxA-34392
R_Date OxA-33175
R_Date OxA-30808
R_Date OxA-28406
R_Date OxA-33176
R_Date OxA-30977
R_Date OxA-30848
R_Date OxA-28392
R_Date ICA-19S/0179
Phase Unit 105
Boundary Start Unit 105
Boundary End Unit 110
Date Unit 110
R_Date OxA-28393
R_Date OxA-28934
Phase Unit 110
Boundary Start Unit 110
Boundary End Unit 111
Date Unit 111
R_Date OxA-28620
Phase Unit 111
Boundary Start Unit 111
Boundary End Unit 107
Date Unit 107
R_Date OxA-28395
R_Date OxA-28408
R_Date OxA-28407
R_Date OxA-34502
R_Date OxA-28409
R_Date OxA-33178
R_Date OxA-33177
R_Date ICA-19S/0180
R_Date OxA-28410
Phase Unit 107
Boundary Start Unit 107
Boundary End Unit 114
Date Unit 114
R_Date OxA-27903
R_Date OxA-27969
Phase Unit 114
Boundary Start Unit 114
Boundary End Unit 115
Date Unit 115
R_Date OxA-31054
R_Date OxA-31055
Phase Unit 115
Boundary Start Unit 115
Boundary End Unit 108
Date Unit 108
R_Date OxA-28396
R_Date OxA-27904
R_Date OxA-31056
R_Date OxA-28411
Phase Unit 108
Boundary Start Unit 108
Sequence
10000
9500
9000
8500
8000
7500
7000
Modelled date (BP)

Supplementary Figure 3. Shell size of a) *Phorcus lineatus* (diameter), b) *Patella vulgate* (length), and c) *Patella depressa* (length) throughout all stratigraphic units of the shell midden site. Red diamonds represent average size for each stratigraphic unit.

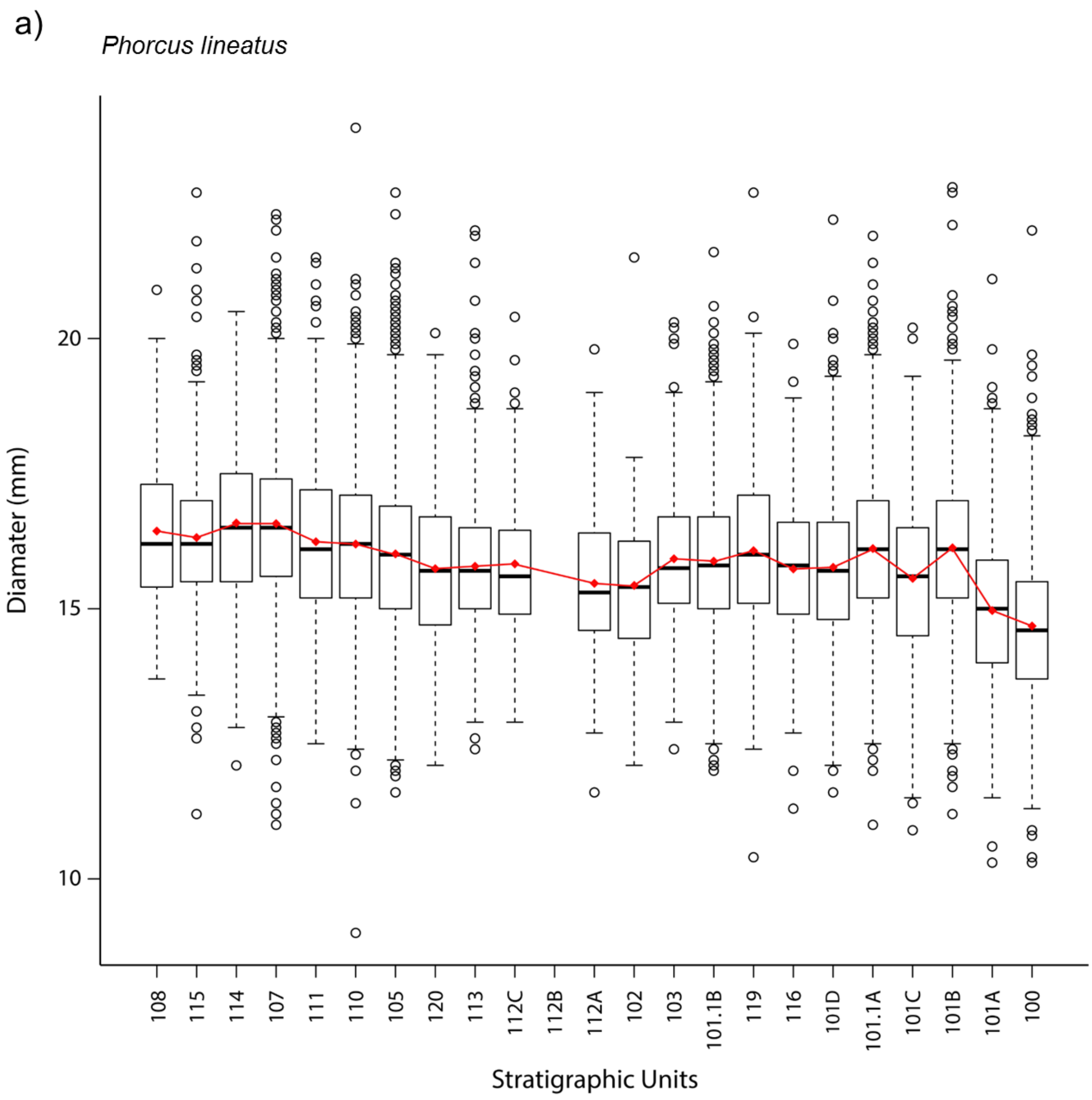

b) *Patella vulgata*

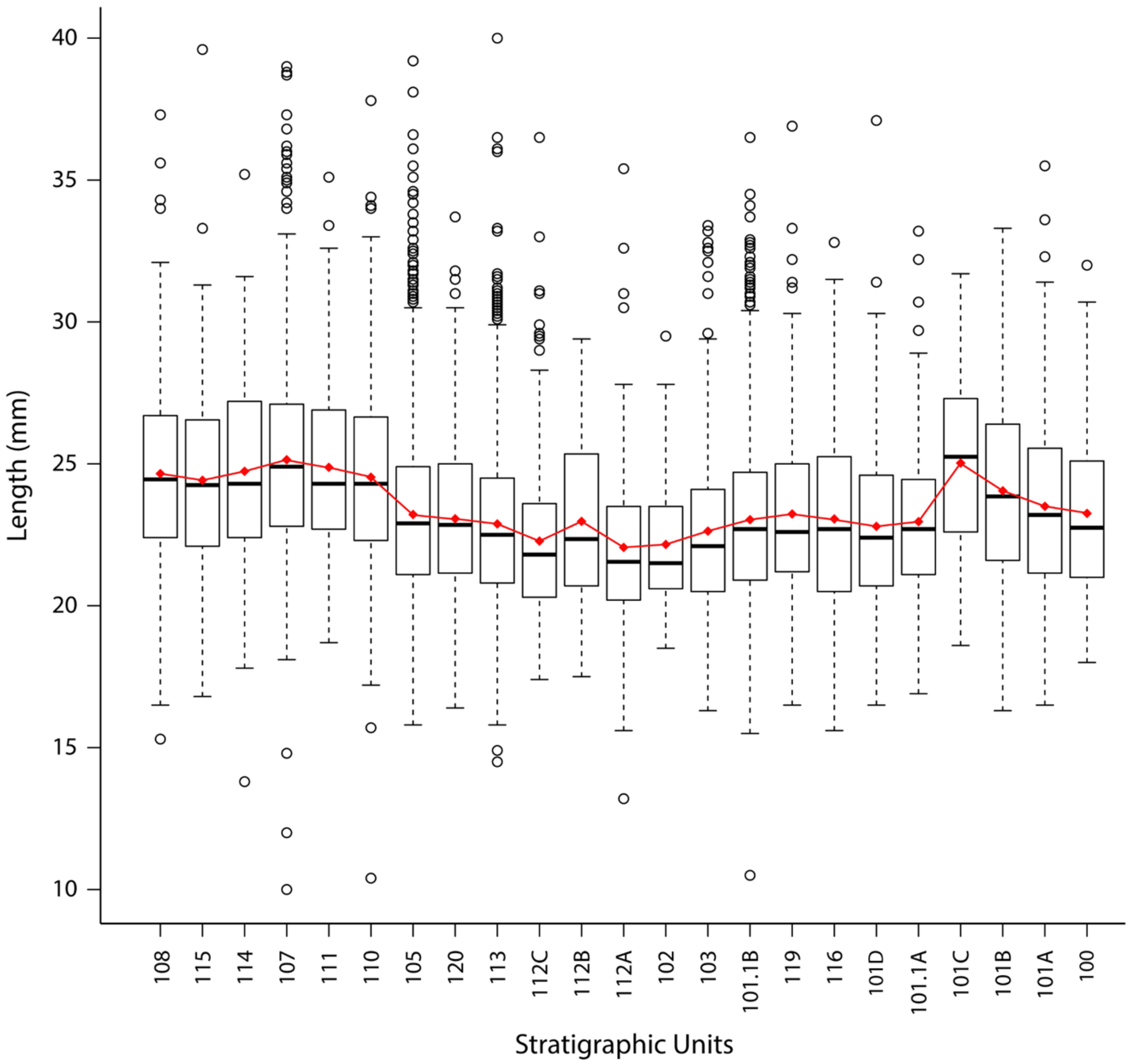

c)

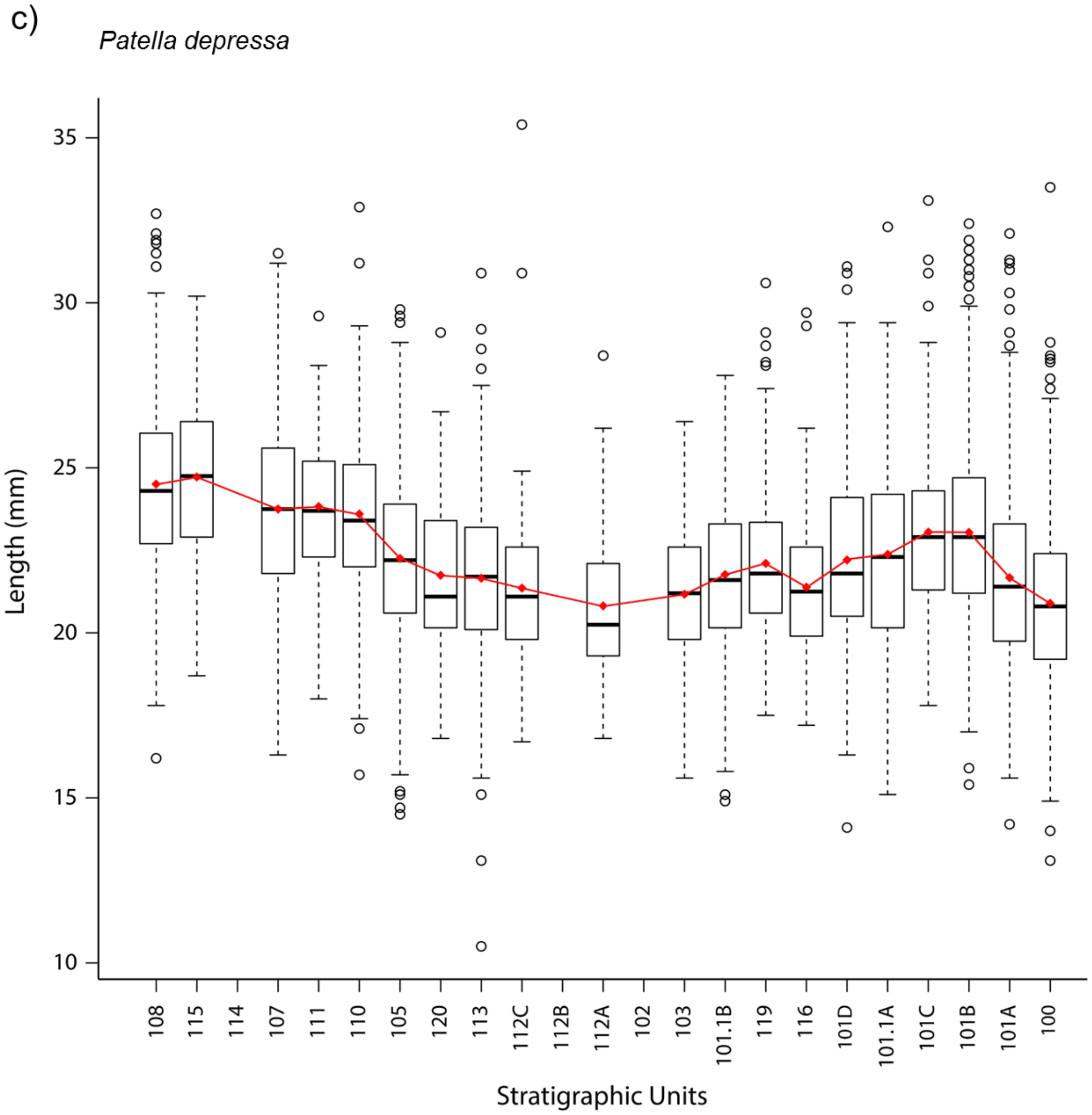

Patella depressa
Length (mm)
35
30
25
20
15
10
108
115
114
107
111
110
105
120
113
112C
112B
112A
102
103
101.1B
119
116
101D
101.1A
101C
101B
101A
100
Stratigraphic Units

Supplementary Figure 4. Early Holocene seasonal insolation evolution in the northern hemisphere (44º N)[15,16] and calculated seasonal SST from $\delta^{18}O_{shell}$ values.

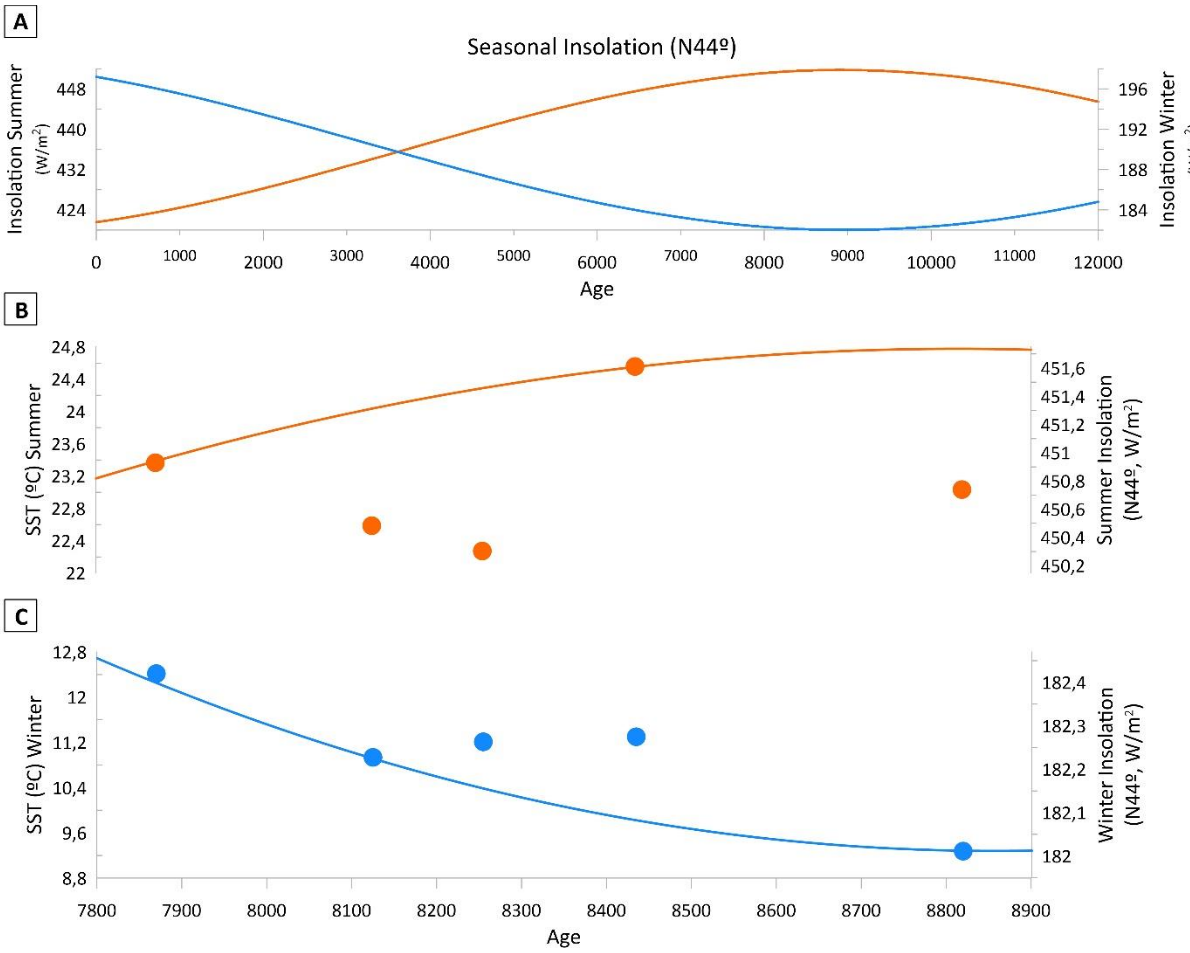

Supplementary Figure 5. Sampling procedures applied to extract calcium carbonate micro-simples from the aragonite layer[6,57,58,59,60]. a) Sampling method used to extract the carbonate from the inner part of the shell aperture. b) Sampling method used to extract the carbonate sequentially along the whorl from the outside of the shell. DoG: Direction of growth.

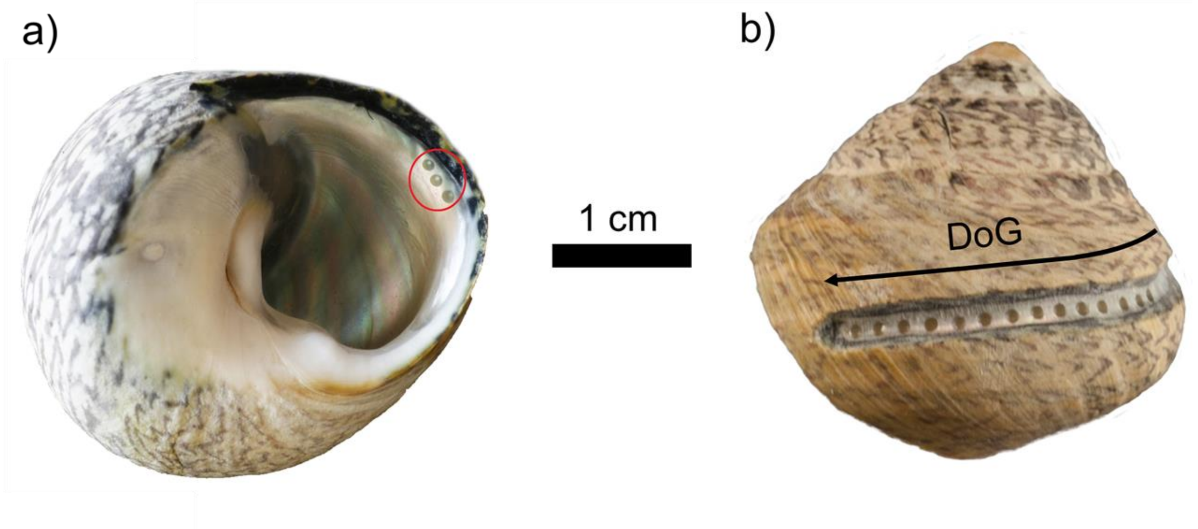

Supplementary Figure 6. Micromorphological thin sections of samples collected in units 101B and 105. Yellow dashed lines indicate the microstratigraphic contacts. In 101B, the different layers within the unit are distinguished by variation in the shell/matrix ratio, as well as by the general burning of the shells in the middle layer (distinguishable by the brighter colour in the dark background scan to the right), which is absent in the top and bottom layers. In 105, the different sedimentary compositions between the layers evinces several colluvial events over the slope, following the erosional surface visible in the profile.

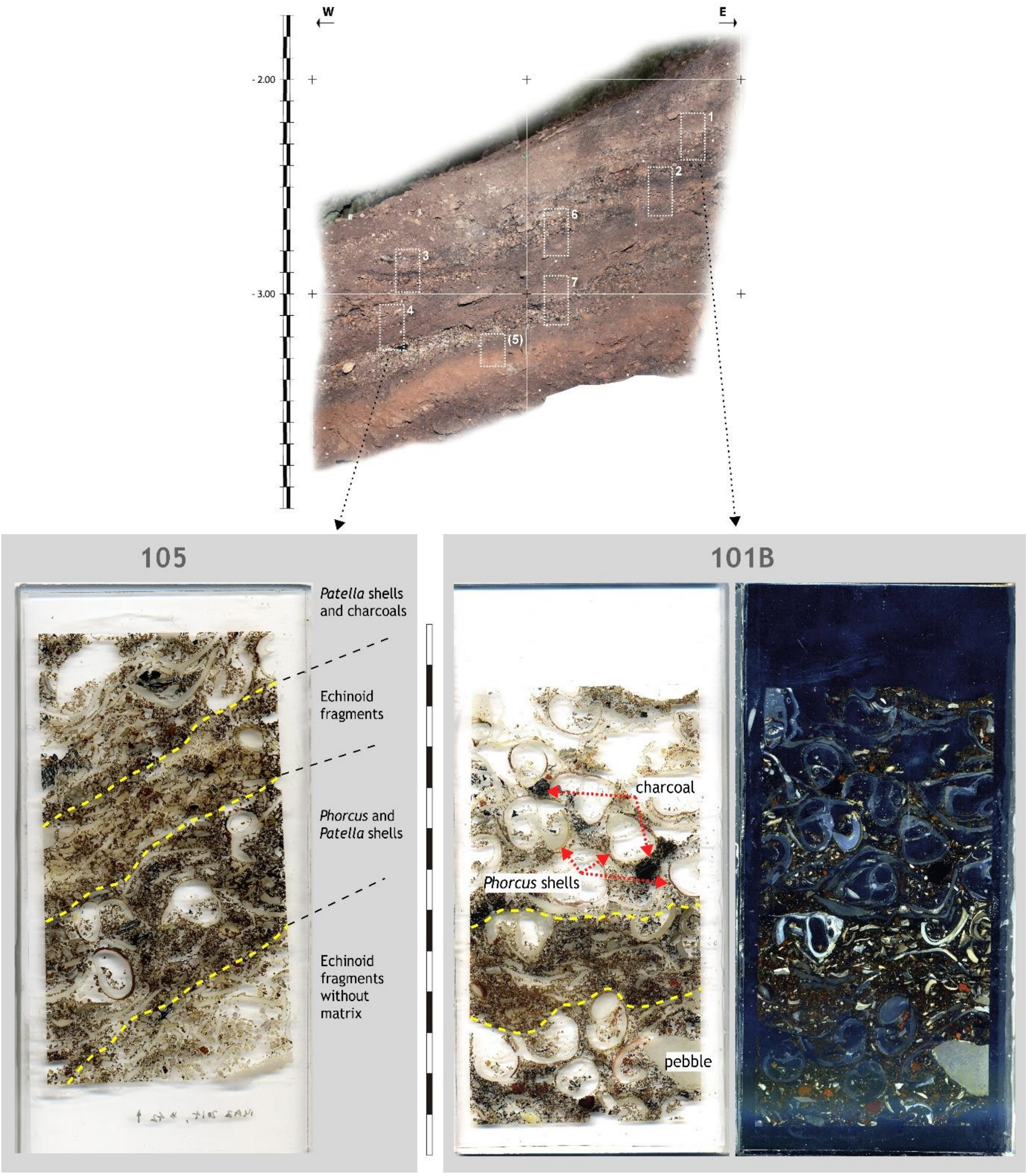

Supplementary Table 1. Radiocarbon determinations for El Mazo cave. This include published and unpublished data as indicated.

Stratigraphic Unit (SU)	Square	Subsquare	Spit	Sample description	^{14}C yrs BP	Laboratory code	Reference
100	X16	D		Bone	6447±40	OxA-30779	Unpublished data
	X15	C		Bone	6450±40	OxA-30807	Unpublished data
	X16	C		Bone	6772±37	OxA-28397	Gutiérrez-Zugasti et al., 2016 [55]
	X15	D		Shell	7212±35	OxA-28387	García-Escárzaga, 2020 [6]
101A	X15	D		Shell	7403±33	OxA-28388	Unpublished data
101B	X15	C		Bone	7105±40	OxA-30780	Soares et al., 2016 [61]
	X15	C		Shell	7310±40	OxA-30806	Soares et al., 2016 [61]
	X15	D		Shell	7475±40	OxA-33171	García-Escárzaga et al., 2022 [62]
	X15	D		Shell	7570±40	OxA-33172	García-Escárzaga et al., 2022 [62]
	X15	D		Shell	7730±40	ICA-19S/0178	García-Escárzaga et al., 2022 [62]
	X15	D		Shell	7786±39	OxA-34390	García-Escárzaga et al., 2022 [62]
101C	X15	D		Bone	7230±36	OxA-28389	Gutiérrez-Zugasti et al., 2016 [55]
101.1A	X15	C		Shell	7357±34	OxA-28390	García-Escárzaga, 2020 [6]
117	X16	D		Charcoal	7159±35	OxA-28400	Unpublished data
101D	X16	D		Bone	7199±36	OxA-28398	Unpublished data
116	X16	C		Shell	7320±30	ICA-18S/0495	Unpublished data
	X16	C		Shell	7501±37	OxA-28399	Unpublished data
119	X15	D	1	Bone	7204±35	OxA-28391	Unpublished data
101.1B	X16	D		Bone	7205±37	OxA-28882	García-Escárzaga, 2020 [6]
	X15	C		Shell	7380±40	ICA-18S/0496	Unpublished data
103	X16	C		Shell	7460±30	ICA-18S/0493	Unpublished data
102	X15	C		Shell	7350±40	ICA-18S/0494	Unpublished data
112A	X16	D		Bone	7294±37	OxA-28401	Gutiérrez-Zugasti et al., 2016 [55]
	X16	D		Bone	7295±40	OxA-31097	Unpublished data
112C	X16	D		Bone	7367±35	OxA-28685	García-Escárzaga et al., 2022 [62]
	X16	D		Shell	7425±34	OxA-28402	García-Escárzaga et al., 2022 [62]
	X16	D		Shell	7480±40	OxA-33173	García-Escárzaga et al., 2019 [57]
	X16	D		Shell	7565±60	OxA-33174	García-Escárzaga, 2020 [6]
	X16	D		Shell	7720±40	ICA-19S/0181	García-Escárzaga et al., 2022 [62]
	X16	D		Shell	7733±38	OxA-34391	García-Escárzaga et al., 2022 [62]
113	X16	C		Charcoal	7212±35	OxA-28403	Soares et al., 2016 [61]
	X16	D		Shell	7492±39	OxA-30849	Unpublished data
	X16	C		Shell	7565±34	OxA-28404	Soares et al., 2016 [61]
120	X16	C		Bone	7412±36	OxA-28405	Soares et al., 2016 [61]
	X16	C		Shell	7625±45	OxA-30976	Soares et al., 2016 [61]

Stratigraphic Unit (SU)	Square	Subsquare	Spit	Sample description	^{14}C yrs BP	Laboratory code	Reference
105	X15	D	2	Charcoal	7380±55	OxA-30535	Soares et al., 2016 [61]
	X16	D	3	Shell	7530±45	OxA-33175	García-Escárzaga et al., 2022 [62]
	X15	D	1	Shell	7540±40	OxA-30808	García-Escárzaga et al., 2022 [62]
	X16	C	1	Shell	7566±34	OxA-28406	García-Escárzaga et al., 2022 [62]
	X16	D	3	Shell	7580±40	OxA-33176	García-Escárzaga, 2020 [6]
	X15	D	2	Shell	7595±40	OxA-30977	Soares et al., 2016 [61]
	X15	C	3	Shell	7609±39	OxA-34392	García-Escárzaga et al., 2022 [62]
	X16	D	3	Shell	7785±40	OxA-30848	García-Escárzaga et al., 2022 [62]
	X15	D	1	Shell	7926±36	OxA-28392	García-Escárzaga et al., 2022 [62]
	X16	D	3	Shell	7980±40	ICA-19S/0179	García-Escárzaga et al., 2022 [62]
110	X15	D	1	Shell	7677±35	OxA-28393	Unpublished data
	X15	C	2	Shell	7717±37	OxA-28934	Unpublished data
111	X15	D	2	Shell	7787±39	OxA-28620	Unpublished data
107	X15	C	3	Charcoal	7438±35	OxA-28395	García-Escárzaga et al., 2022 [62]
	X16	D	1	Bone	7618±37	OxA-28408	García-Escárzaga et al., 2019 [57]
	X16	D	1	Charcoal	7694±36	OxA-28407	García-Escárzaga et al., 2022 [62]
	X16	D	1	Shell	7681±34	OxA-28409	García-Escárzaga et al., 2022 [62]
	X16	D	1	Shell	7730±40	OxA-33178	García-Escárzaga et al., 2022 [62]
	X16	D	1	Shell	7805±40	OxA-33177	García-Escárzaga, 2020 [6]
	X16	D	1	Shell	7870±40	ICA-19S/0180	García-Escárzaga et al., 2022 [62]
	X16	C	1	Shell	7929±35	OxA-28410	García-Escárzaga et al., 2022 [62]
	X16	D	1	Shell	7935±38	OxA-34502	García-Escárzaga et al., 2022 [62]
114	X16	C		Shell	7538±34	OxA-27903	Unpublished
	X16	C		Bone	7990±38	OxA-27969	Gutiérrez-Zugasti et al., 2016 [55]
115	X16	C	2	Bone	8000±40	OxA-31054	García-Escárzaga et al., 2017 [56]
	X16	C	2	Bone	8004±39	OxA-31055	García-Escárzaga et al., 2017 [56]
108	X15	D		Shell	7935±35	OxA-28396	García-Escárzaga et al., 2015 [63]
	X16	C		Shell	8222±36	OxA-27904	Soares et al., 2016 [61]
	X15	C		Bone	8040±40	OxA-31056	Unpublished data
	X16	C		Bone	8022±39	OxA-28411	Soares et al., 2016 [61]

Supplementary Table 2. Sample description of every sample radiocarbon dated. Table includes yield and C:N ratio values.

Stratigraphic Unit (SU)	Laboratory code	Sample description	Taxa	Yield	C:N ratio	Reference
100	OxA-30779	Bone	Middle size Ungulate	13.21	3.25	Unpublished data
	OxA-30807	Bone	*Sus* sp.	2.8	3.71	Unpublished data
	OxA-28397	Bone	Young Mid-sized Mammal	1.7	3.29	Gutiérrez-Zugasti et al., 2016 [55]
	OxA-28387	Shell	*Phorcus lineatus*	10.4		García-Escárzaga, 2020 [6]
101A	OxA-28388	Shell	*Phorcus lineatus*	9.8		Unpublished data
101B	OxA-30780	Bone	*Capreolus capreolus*	6.9	3.17	Soares et al., 2016 [61]
	OxA-30806	Shell	*Phorcus lineatus*	10.4		Soares et al., 2016 [61]
	OxA-33171	Shell	*Phorcus lineatus*	10.8		García-Escárzaga et al., 2022 [62]
	OxA-33172	Shell	*Phorcus lineatus*	10.8		García-Escárzaga et al., 2022 [62]
	ICA-19S/0178	Shell	*Phorcus lineatus*			García-Escárzaga et al., 2022 [62]
	OxA-34390	Shell	*Patella vulgata*	10.6		García-Escárzaga et al., 2022 [62]
101C	OxA-28389	Bone	Ungulate middle size	4	3.31	Gutiérrez-Zugasti et al., 2016 [55]
101.1A	OxA-28390	Shell	*Phorcus lineatus*	10.1		García-Escárzaga, 2020 [6]
117	OxA-28400	Charcoal	*Corylus avellana*	9.4		Unpublished data
101D	OxA-28398	Bone	Ungulate middle size	7.7	3.32	Unpublished data
116	ICA-18S/0495	Shell	*Phorcus lineatus*			Unpublished data
	OxA-28399	Shell	*Phorcus lineatus*	9.2		Unpublished data
119	OxA-28391	Bone	*Cervus elaphus*	2.4	3.32	Unpublished data
101.1B	OxA-28882	Bone	*Capreolus capreolus*	9.5	3.37	García-Escárzaga, 2020 [6]
	ICA-18S/0496	Shell	*Phorcus lineatus*			Unpublished data
103	ICA-18S/0493	Shell	*Phorcus lineatus*			Unpublished data
102	ICA-18S/0494	Shell	*Phorcus lineatus*			Unpublished data
112A	OxA-28401	Bone	*Cervus elaphus*	2	3.3	Gutiérrez-Zugasti et al., 2016 [55]
	OxA-31097	Bone	*Cervus elaphus*	8.8	3.28	Unpublished data
112C	OxA-28685	Bone	*Capreolus capreolus*	6	3.26	García-Escárzaga et al., 2022 [62]
	OxA-28402	Shell	*Phorcus lineatus*	10		García-Escárzaga et al., 2022 [62]
	OxA-33173	Shell	*Phorcus lineatus*	11.1		García-Escárzaga et al., 2019 [57]
	OxA-33174	Shell	*Phorcus lineatus*	11		García-Escárzaga, 2020 [6]
	ICA-19S/0181	Shell	*Phorcus lineatus*			García-Escárzaga et al., 2022 [62]
	OxA-34391	Shell	*Patella vulgata*	11		García-Escárzaga et al., 2022 [62]
113	OxA-28403	Charcoal	*Corylus avellana*	17.5		Soares et al., 2016 [61]
	OxA-30849	Shell	*Phorcus lineatus*	9.3		Unpublished data
	OxA-28404	Shell	*Phorcus lineatus*	9.7		Soares et al., 2016 [61]
120	OxA-28405	Bone	Ungulate	9	3.31	Soares et al., 2016 [61]
	OxA-30976	Shell	*Phorcus lineatus*	10.4		Soares et al., 2016 [61]

Stratigraphic Unit (SU)	Laboratory code	Sample description	Taxa	Yield	C:N ratio	Reference
105	OxA-30535	Charcoal	*Corylus avellana*	17.7		Soares et al., 2016 [61]
	OxA-33175	Shell	*Phorcus lineatus*	10.9		García-Escárzaga et al., 2022 [62]
	OxA-30808	Shell	*Phorcus lineatus*	10.4		García-Escárzaga et al., 2022 [62]
	OxA-28406	Shell	*Phorcus lineatus*			García-Escárzaga et al., 2022 [62]
	OxA-33176	Shell	*Phorcus lineatus*	8.8		García-Escárzaga, 2020 [6]
	OxA-30977	Shell	*Phorcus lineatus*	10.5		Soares et al., 2016 [61]
	OxA-34392	Shell	*Patella vulgata*	11		García-Escárzaga et al., 2022 [62]
	OxA-30848	Shell	*Phorcus lineatus*	9		García-Escárzaga et al., 2022 [62]
	OxA-28392	Shell	*Phorcus lineatus*	9.9		García-Escárzaga et al., 2022 [62]
	ICA-19S/0179	Shell	*Phorcus lineatus*			García-Escárzaga et al., 2022 [62]
110	OxA-28393	Shell	*Phorcus lineatus*	7.3		Unpublished data
	OxA-28934	Shell	*Phorcus lineatus*	9.1		Unpublished data
111	OxA-28620	Shell	*Phorcus lineatus*	9.8		Unpublished data
107	OxA-28395	Charcoal	*Corylus avellana*	22		García-Escárzaga et al., 2022 [62]
	OxA-28408	Bone	Middle size Ungulate	1.1	3.29	García-Escárzaga et al., 2019 [57]
	OxA-28407	Charcoal	*Quercus*	40		García-Escárzaga et al., 2022 [62]
	OxA-28409	Shell	*Phorcus lineatus*	3.1		García-Escárzaga et al., 2022 [62]
	OxA-33178	Shell	*Phorcus lineatus*	8.8		García-Escárzaga et al., 2022 [62]
	OxA-33177	Shell	*Phorcus lineatus*	10.6		García-Escárzaga, 2020 [6]
	ICA-19S/0180	Shell	*Phorcus lineatus*			García-Escárzaga et al., 2022 [62]
	OxA-28410	Shell	*Phorcus lineatus*	9.8		García-Escárzaga et al., 2022 [62]
	OxA-34502	Shell	*Patella vulgata*	11		García-Escárzaga et al., 2022 [62]
114	OxA-27903	Shell	*Phorcus lineatus*	10.4		Unpublished
	OxA-27969	Bone	*Capra* sp./*Capreolus capreolus*	8.3	3.31	Gutiérrez-Zugasti et al., 2016 [55]
115	OxA-31054	Bone	*Cervus elaphus*	8.7	3.0995	García-Escárzaga et al., 2017 [56]
	OxA-31055	Bone	*Cervus elaphus*	8.9	3.099	García-Escárzaga et al., 2017 [56]
108	OxA-28396	Shell	*Phorcus lineatus*	9.8		García-Escárzaga et al., 2015 [63]
	OxA-27904	Shell	*Phorcus lineatus*	10.5		Soares et al., 2016 [61]
	OxA-31056	Bone	Middle size Ungulate	1.2	3.26	Unpublished data
	OxA-28411	Bone	*Capreolus capreolus*	0.8	3.294	Soares et al., 2016 [61]

Supplementary Table 3. Minimum number of individuals (MNI) for each mollusc taxa in each stratigraphic unit (SU).

Taxa \ SU	108	115	114	107	111	110	105	120	113	112C	112B	112A
**Marine bivalve**												
Mytilus galloprincialis	12	34	4	57	2	12	243	28	122	8	1	1
Ruditapes decussatus	0	0	0	0	0	0	0	0	0	1	1	1
**Total Bivalves**	**12**	**34**	**4**	**57**	**2**	**12**	**243**	**28**	**122**	**9**	**2**	**2**
**Marine gastropods**												
Bittium latreillii	0	1	0	0	0	0	0	0	0	0	0	0
Bittium reticulatum	0	0	0	0	0	0	5	0	2	0	0	0
Calliostoma zizyphinum	0	0	0	0	0	0	1	0	0	0	0	0
Charonia lampas	0	0	0	0	0	0	1	0	0	0	0	0
Haliotis tuberculata	0	0	0	0	0	0	0	0	2	0	0	0
Littorina obtusata	0	0	0	1	1	0	3	0	3	0	0	0
Littorina saxatilis	1	0	0	0	0	0	14	1	9	0	1	1
Melaraphe neritoides	0	0	2	4	2	5	28	0	2	1	0	0
Nucella lapillus	0	1	0	2	0	1	7	0	7	0	0	0
Ocenebra erinaceus	0	0	0	0	0	0	0	0	0	0	0	0
Ocinebrina sp.	0	0	0	0	0	2	3	0	0	0	0	0
Patella vulgata	536	297	87	950	189	801	4530	695	2852	402	137	315
Patella depressa	230	96	9	356	66	339	1318	112	516	108	30	100
Patella ulyssiponensis	35	10	2	14	5	13	179	29	137	16	3	15
Patella sp.	855	414	62	930	227	591	4565	419	1255	258	79	158
Phorcus lineatus	329	1000	217	4140	682	3516	5595	529	1574	207	44	213
Steromphala sp.	16	12	6	27	7	32	98	15	28	4	0	0
Tritia incrassata	0	0	0	7	0	0	0	0	0	0	0	0
Tritia reticulata	1	1	0	1	3	2	15	1	3	0	0	0
Trivia sp.	0	0	0	0	0	0	0	0	0	0	0	0
**Total Mar. gastropods**	**2003**	**1832**	**385**	**6432**	**1182**	**5302**	**16362**	**1801**	**6390**	**996**	**294**	**802**
**Scaphopods**												
Antalis novemcostata	0	0	0	0	0	0	0	0	0	0	0	0
**TOTAL**	**2015**	**1866**	**389**	**6489**	**1184**	**5314**	**16605**	**1829**	**6512**	**1005**	**296**	**804**

Taxa \ SU	102	103	101.1B	119	116	101D	101.1A	101C	101B	101A	100
**Marine bivalve**											
Mytilus galloprincialis	14	7	10	9	3	47	16	8	24	35	176
Ruditapes decussatus	2	1	1	1	0	2	0	0	1	0	0
**Total Bivalves**	**16**	**8**	**11**	**10**	**3**	**49**	**16**	**8**	**25**	**35**	**176**
**Marine gastropods**											
Bittium latreillii	0	0	0	0	0	0	0	0	0	0	0
Bittium reticulatum	0	1	0	0	0	2	0	1	3	2	4
Calliostoma zizyphinum	0	0	0	0	0	0	0	7	0	0	0
Charonia lampas	0	0	0	0	0	0	0	0	0	0	0
Haliotis tuberculata	0	0	1	1	1	1	2	3	8	3	5
Littorina obtusata	0	1	1	1	0	0	1	0	0	0	0
Littorina saxatilis	0	0	2	1	0	4	3	1	5	0	1
Melaraphe neritoides	0	0	5	0	0	3	2	0	10	8	12
Nucella lapillus	1	0	0	1	0	1	0	0	0	1	0
Ocenebra erinaceus	0	0	0	0	0	0	0	0	0	0	0
Ocinebrina sp.	0	0	0	0	0	0	0	0	0	0	0
Patella vulgata	100	618	2480	254	227	479	406	160	411	380	268
Patella depressa	26	181	679	202	97	467	356	396	915	792	1042
Patella ulyssiponensis	1	25	71	59	7	50	78	78	213	123	243
Patella sp.	1005	354	1517	901	199	708	502	267	681	849	1998
Phorcus lineatus	348	531	2157	800	470	1789	1846	1036	2241	1687	1565
Steromphala sp.	0	0	17	3	3	9	3	9	9	18	7
Tritia incrassata	0	0	0	0	0	0	0	0	0	0	0
Tritia reticulata	1	0	1	0	0	2	1	3	8	5	6
Trivia sp.	0	0	1	0	0	1	0	2	0	1	0
**Total Mar. gastropods**	**1482**	**1711**	**6932**	**2223**	**1004**	**3516**	**3200**	**1963**	**4504**	**3869**	**5151**
**Scaphopods**											
Antalis novemcostata	0	0	0	0	0	1	0	0	0	0	0
**TOTAL**	**1498**	**1719**	**6943**	**2233**	**1007**	**3566**	**3216**	**1971**	**4529**	**3904**	**5327**

Supplementary Table 4. Number of *P. lineatus*, *P. vulgata* and *P. depressa* specimens measured for biometric analysis from each stratigraphic unit.

Stratigraphic unit	Number of specimens measured		
	P. lineatus	*P. vulgata*	*P. depressa*
**100**	846	105	423
**101A**	1017	195	468
**101B**	1382	230	523
**101C**	690	98	212
**101.1A**	864	223	183
**101D**	924	290	229
**116**	241	116	58
**119**	364	174	152
**101.1B**	1544	1417	383
**103**	238	383	113
**102**	39	51	12
**112A**	105	186	50
**112B**	25	84	13
**112C**	100	249	69
**113**	989	1578	270
**120**	325	444	71
**105**	3181	3161	637
**110**	2466	475	186
**111**	625	110	36
**107**	3238	844	238
**114**	158	51	5
**115**	707	176	50
**108**	176	392	168
**Total**	**20244**	**11032**	**4549**

Supplementary Table 5. Statistical results derived from Shapiro-Wilk (p-value) and univariate (Skewness value) tests for a) *Phorcus lineatus*, b) *Patella vulgata* and c) *Patella depressa*. Units that showed a normal or log-normal shell size distribution are shown with a grey background.

a)

Phocus lineatus				
**Stratigraphic unit**	**n**	**Diameter Shapiro-Wilk (p-value)**		**Skewness**
		Normal	Log-Normal	
**100**	846	< 0,0001	0.002	0.57
**101A**	1017	0.02	**0.25**	0.2
**101B**	1382	< 0,0001	< 0,0001	0.22
**101C**	690	**0.11**		0.17
**101.1A**	864	0.0002	0.01	0.26
**101D**	924	0.0002	**0.55**	0.05
**116**	241	**0.36**	0.02	-0.03
**119**	364	0.003	0.007	0.31
**101.1B**	1544	< 0,0001	**0.15**	0.33
**103**	238	0.0001	0.02	0.59
**102**	39	**0.18**		-0.3
**112A**	105	**0.19**		0.34
**112B**		-----	-----	-----
**112C**	100	0.0008	0.03	0.8
**113**	989	< 0,0001	< 0,0001	0.7
**120**	325	**0.07**		0.31
**105**	3181	< 0,0000	0.01	0.31
**110**	2466	< 0,0001	< 0,0001	0.12
**111**	625	< 0,0001	**0.21**	0.33
**107**	3238	< 0,0001	0.0001	0.23
**114**	158	**0.67**		0.11
**115**	707	< 0,0001	< 0,0001	0.57
**108**	176	0.0021	0.045	0.51

b)	*Patella vulgata*			
**Stratigraphic unit**	**n**	**Length Shapiro-Wilk (p-value)**		**Skewness**
		Normal	Log-Normal	
**100**	105	0.006	**0.1**	0.6
**101A**	195	0.02	**0.78**	0.5
**101B**	230	**0.15**		0.29
**101C**	98	**0.14**		-0.01
**101.1A**	223	0.0003	**0.35**	0.7
**101D**	290	< 0,0001	0.02	0.85
**116**	116	0.009	**0.42**	0.56
**119**	174	< 0,0001	0.001	1.15
**101.1B**	1417	< 0,0001	< 0,0001	0.64
**103**	383	< 0,0001	< 0,0001	0.99
**102**	51	0.009	0.96	0.89
**112A**	186	< 0,0001	0.006	0.91
**112B**	84	0.04	**0.23**	0.31
**112C**	249	0.004	< 0,0001	1.19
**113**	1578	< 0,0001	< 0,0001	0.8
**120**	444	0.005	**0.75**	0.37
**105**	3161	< 0,0000	< 0,0001	0.77
**110**	475	< 0,0001	< 0,0001	0.34
**111**	110	0.04	**0.6**	0.57
**107**	844	< 0,0001	< 0,0001	0.54
**114**	51	**0.76**		0.09
**115**	176	0.001		0.64
**108**	392	0.003		0.43

c)

Patella depressa				
**Stratigraphic unit**	**n**	**Length Shapiro-Wilk (p-value)**		**Skewness**
		Normal	Log-Normal	
**100**	423	< 0,0001	**0.07**	0.53
**101A**	468	< 0,0001	**0.09**	0.67
**101B**	523	< 0,0001	**0.26**	0.44
**101C**	212	0.0005	**0.23**	0.67
**101.1A**	183	**0.07**		0.42
**101D**	229	0.03	**0.16**	0.34
**116**	58	0.006	**0.14**	0.97
**119**	152	0.0006	**0.11**	0.79
**101.1B**	383	**0.66**		0.08
**103**	113	**0.95**		0.01
**102**		-----	-----	-----
**112A**	50	0.03	**0.27**	0.97
**112B**		-----	-----	-----
**112C**	69	< 0,0001	0.03	2.31
**113**	270	0.009	< 0,0001	-0.08
**120**	71	**0.28**		0.47
**105**	637	**0.08**	0.01	0.15
**110**	186	**0.07**	0.03	0.21
**111**	36	**0.99**		0.06
**107**	238	**0.77**		0.16
**114**		-----	-----	-----
**115**	50	**0.99**		-0.08
**108**	168	**0.15**		0.29

Supplementary Table 6. Percentage of specimens of *Patella vulgata* species collected from exposed and sheltered shores. Results were obtained using the regression equation published by Bailey and Craighead[22].

Stratigraphic unit	Exposed coast	Sheltered coast
**100**	98.9	1.1
**101A**	98.9	1.1
**101B**	99.1	0.9
**101C**	100	0
**101.1A**	99.1	0.9
**101D**	99.6	0.4
**116**	99.1	0.9
**119**	96.9	3.1
**101.1B**	99.3	0.7
**103**	97.4	2.6
**102**	95.8	4.2
**112A**	98.8	1.2
**112B**	97.4	2.6
**112C**	98.3	1.7
**113**	99.4	0.6
**120**	99.3	0.7
**105**	99.1	0.9
**110**	97.9	2.1
**111**	94.9	5.1
**107**	96.1	3.9
**114**	95.7	4.3
**115**	98.7	1.3
**108**	97.9	2.1

Supplementary Code 1. OxCal 4.4 CQL code for Supplementary Figure 2. Bayesian age model (El Mazo Cave).

```
Plot()
 {
  Outlier_Model("General",T(5),U(0,4),"t");
  Curve("IntCal20","IntCal20.14c");
  Curve("Marine20","Marine20.14c");
  Sequence()
  {
   Boundary("Start Unit 108");
   Phase("Unit 108")
   {
    Curve("=IntCal20");
R_Date("OxA-28411", 8022, 39)
    {
     Outlier("General", 0.05);
    };
R_Date("OxA-31056", 8040, 40)
    {
     Outlier("General", 0.05);
    };
Curve("=Marine20");
    Delta_R("Phorcus 108",-257, 120);
    R_Date("OxA-27904", 8222, 36)
    {
     Outlier("General", 0.05);
    };


    R_Date("OxA-28396", 7935, 35)
    {
     Outlier("General", 0.05);
    };
Interval("Duration of Unit 108");
```

```
Date("Sample from Unit 108");
   };
 Boundary("End Unit 108");
Boundary("Start Unit 115");
   Phase("Unit 115")
   {
    Curve("=IntCal20");
    R_Date("OxA-31055", 8004, 39)
    {
     Outlier("General", 0.05);
    };
R_Date("OxA-31054", 8000, 40)
    {
     Outlier("General", 0.05);
    };
Interval("Duration of Unit 115");
Date("Sample from Unit 115");
   };
   Boundary("End Unit 115");
Boundary("Start Unit 114");
   Phase("Unit 114")
   {
    Curve("=IntCal20");
    R_Date("OxA-27969", 7990, 38)
    {
     Outlier("General", 0.05);
    };
Curve("=Marine20");
    Delta_R("Phorcus 114",-257, 120);
    R_Date("OxA-27903", 7538, 34)
    {
     Outlier("General", 0.05);
    };
    Interval("Duration of Unit 114");
```

```
  Date("Sample from Unit 114");
  };
    Boundary("End Unit 114");
Boundary("Start Unit 107");
  Phase("Unit 107")
  {
   Curve("=Marine20");
   Delta_R("Phorcus 107",-257, 120);
   R_Date("OxA-28410", 7929, 35)
   {
    Outlier("General", 0.05);
   };
   R_Date("ICA-19S/0180", 7870, 40)
   {
    Outlier("General", 0.05);
   };
   R_Date("OxA-33177", 7805, 40)
   {
    Outlier("General", 0.05);
   };
   R_Date("OxA-33178", 7730, 40)
   {
    Outlier("General", 0.05);
   };
   R_Date("OxA-28409", 7681, 34)
   {
    Outlier("General", 0.05);
   };
   Curve("=Marine20");
   Delta_R("Patella 107",-114, 170);
   R_Date("OxA-34502", 7935, 38)
   {
    Outlier("General", 0.05);
   };
```

```
    Curve("=IntCal20");
    R_Date("OxA-28407", 7694, 36)
    {
     Outlier("General", 0.05);
    };
R_Date("OxA-28408", 7618, 37)
    {
     Outlier("General", 0.05);
    };
R_Date("OxA-28395", 7438, 35)
    {
     Outlier("General", 0.05);
    };
    Interval("Duration of Unit 107");
    Date("Sample from Unit 107");
};
      Boundary("End Unit 107");
Boundary("Start Unit 111");
Phase("Unit 111")
   {
Curve("=Marine20");
    Delta_R("Phorcus 111",-257, 120);
    R_Date("OxA-28620", 7787, 39)
    {
     Outlier("General", 0.05);
    };
    Interval("Duration of Unit 111");
    Date("Sample from Unit 111");
   };
      Boundary("End Unit 111");
Boundary("Start Unit 110");
Phase("Unit 110")
   {
       Curve("=Marine20");
```

```
    Delta_R("Phorcus 110",-257, 120);
    R_Date("OxA-28394", 7717, 37)
    {
     Outlier("General", 0.05);
    };
    R_Date("OxA-28393", 7677, 35)
    {
     Outlier("General", 0.05);
    };
    Interval("Duration of Unit 110");
    Date("Sample from Unit 110");
   };
      Boundary("End Unit 110");
Boundary("Start Unit 105");
Phase("Unit 105")
   {
    Curve("=Marine20");
    Delta_R("Phorcus 105",-257, 120);
    R_Date("ICA-19S/0179", 7980, 40)
    {
     Outlier("General", 0.05);
    };
    R_Date("OxA-28392", 7926, 36)
    {
     Outlier("General", 0.05);
    };
    R_Date("OxA-30848", 7785, 40)
    {
     Outlier("General", 0.05);
    };
    R_Date("OxA-30977", 7595, 40)
    {
     Outlier("General", 0.05);
    };
```

```
    R_Date("OxA-33176", 7580, 40)
    {
     Outlier("General", 0.05);
    };
    R_Date("OxA-28406", 7566, 34)
    {
     Outlier("General", 0.05);
    };
    R_Date("OxA-30808", 7540, 40)
    {
     Outlier("General", 0.05);
    };
    R_Date("OxA-33175", 7530, 45)
    {
     Outlier("General", 0.05);
    };
    Curve("=Marine20");
    Delta_R("Patella 105",-114, 170);
    R_Date("OxA-34392", 7609, 39)
    {
     Outlier("General", 0.05);
    };
    Curve("=IntCal20");
    R_Date("OxA-30535", 7380, 55)
    {
     Outlier("General", 0.05);
    };
    Interval("Duration of Unit 105");
    Date("Sample from Unit 105");
   };
        Boundary("End Unit 105");
Boundary("Start Unit 120");
   Phase("Unit 120")
   {
```

```
    Curve("=Marine20");
    Delta_R("Phorcus 120",-257, 120);
    R_Date("OxA-30976", 7625, 45)
    {
     Outlier("General", 0.05);
    };
    Curve("=IntCal20");
    R_Date("OxA-28405", 7412, 36)
    {
     Outlier("General", 0.05);
    };
    Interval("Duration of Unit 120");
    Date("Sample from Unit 120");
   };
         Boundary("End Unit 120");
Boundary("Start Unit 113");
   Phase("Unit 113")
   {
    Curve("=Marine20");
    Delta_R("Phorcus 113",-257, 120);
    R_Date("OxA-28404", 7565, 34)
    {
     Outlier("General", 0.05);
    };
    R_Date("OxA-30849", 7492, 39)
    {
     Outlier("General", 0.05);
    };
    Curve("=IntCal20");
    R_Date("OxA-28403", 7212, 35)
    {
     Outlier("General", 0.05);
    };
        Interval("Duration of Unit 113");
```

```
        Date("Sample from Unit 113");
    };
                Boundary("End Unit 113");
Boundary("Start Unit 112C");
  Phase("Unit 112C")
  {
   Curve("=IntCal20");
   R_Date("OxA-28685", 7367, 35)
   {
    Outlier("General", 0.05);
   };
   Curve("=Marine20");
   Delta_R("Phorcus 112C",-257, 120);
   R_Date("ICA-19S/0181", 7720, 40)
   {
    Outlier("General", 0.05);
   };
   R_Date("OxA-33174", 7565, 60)
   {
    Outlier("General", 0.05);
   };
   R_Date("OxA-33173", 7480, 40)
   {
    Outlier("General", 0.05);
   };
   R_Date("OxA-28402", 7425, 34)
   {
    Outlier("General", 0.05);
   };
   Curve("=Marine20");
   Delta_R("Patella 112C",-114, 170);
   R_Date("OxA-34391", 7733, 38)
   {
    Outlier("General", 0.05);
```

```
  };
  Interval("Duration of Unit 112C");
  Date("Sample from Unit 112C");
 };
        Boundary("End Unit 112C");
Boundary("Start Unit 112A");
 Phase("Unit 112A")
 {
  Curve("=IntCal20");
  R_Date("OxA-31097", 7295, 40)
  {
   Outlier("General", 0.05);
  };
  R_Date("OxA-28401", 7294, 37)
  {
   Outlier("General", 0.05);
  };
  Interval("Duration of Unit 112A");
  Date("Sample from Unit 112A");
 };
Boundary("End Unit 112A");
Boundary("Start Unit 102");
 Phase("Unit 102")
 {
  Curve("=Marine20");
  Delta_R("Phorcus 102",-257, 120);
  R_Date("ICA-18S/0494", 7350, 40)
  {
   Outlier("General", 0.05);
  };

  Interval("Duration of Unit 102");
  Date("Sample from Unit 102");
 };
```

```
   Boundary("End Unit 102");
Boundary("Start Unit 103");
   Phase("Unit 103")
   {
    Curve("=Marine20");
    Delta_R("Phorcus 103",-257, 120);
    R_Date("ICA-18S/0493", 7460, 30)
    {
     Outlier("General", 0.05);
    };

        Interval("Duration of Unit 103");
        Date("Sample from Unit 103");
   };
Boundary("End Unit 103");
Boundary("Start Unit 101.1B");
   Phase("Unit 101.1B")
   {
    Curve("=IntCal20");
    R_Date("OxA-28982", 7205, 37)
      {
     Outlier("General", 0.05);
    };
    Curve("=Marine20");
    Delta_R("Phorcus 101.1B",-257, 120);
    R_Date("ICA-18S/0496", 7380, 40)
    {
     Outlier("General", 0.05);
    };
        Interval("Duration of Unit 101.1B");
        Date("Sample from Unit 101.1B");
   };
   Boundary("End Unit 101.1B");
Boundary("Start Unit 119");
```

```
  Phase("Unit 119")
  {
   Curve("=IntCal20");
   R_Date("OxA-28391", 7204, 35)
   {
    Outlier("General", 0.05);
   };
      Interval("Duration of Unit 119");
      Date("Sample from Unit 119");
  };
  Boundary("End Unit 119");
Boundary("Start Unit 116");
  Phase("Unit 116")
  {
   Curve("=Marine20");
   Delta_R("Phorcus 116",-257, 120);
   R_Date("OxA-28399", 7501, 37)
   {
    Outlier("General", 0.05);
   };
   R_Date("ICA-18S/0495", 7320, 30)
   {
    Outlier("General", 0.05);
   };
      Interval("Duration of Unit 116");
      Date("Sample from Unit 116");
  };
  Boundary("End Unit 116");
Boundary("Start Unit 101D");
  Phase("Unit 101D")
  {
   Curve("=IntCal20");
   R_Date("OxA-28398", 7199, 36)
   {
```

```
     Outlier("General", 0.05);
    };
       Interval("Duration of Unit 101D");
       Date("Sample from Unit 101D");
   };
   Boundary("End Unit 101D");
Boundary("Start Unit 117");
   Phase("Unit 117")
   {
    Curve("=IntCal20");
    R_Date("OxA-28400", 7159, 35)
    {
     Outlier("General", 0.05);
    };
       Interval("Duration of Unit 117");
       Date("Sample from Unit 117");
   };
   Boundary("End Unit 117");
Boundary("Start Unit 101.1A");
   Phase("Unit 101.1A")
   {
    Curve("=Marine20");
    Delta_R("Phorcus 101.1A",-257, 120);
    R_Date("OxA-28390", 7357, 34)
    {
     Outlier("General", 0.05);
    };
       Interval("Duration of Unit 101.1A");
       Date("Sample from Unit 101.1A");
   };
   Boundary("End Unit 101.1A");
Boundary("Start Unit 101C");
   Phase("Unit 101C")
   {
```

```
  Curve("=IntCal20");
  R_Date("OxA-28389", 7230, 36)
  {
   Outlier("General", 0.05);
  };
  Interval("Duration of Unit 101C");
  Date("Sample from Unit 101C");
 };
 Boundary("End Unit 101C");
Boundary("Start Unit 101B");
 Phase("Unit 101B")
 {
  Curve("=Marine20");
  Delta_R("Phorcus 101B",-257, 120);
  R_Date("OxA-33171", 7475, 40)
  {
   Outlier("General", 0.05);
  };
  R_Date("OxA-33172", 7570, 40)
  {
   Outlier("General", 0.05);
  };
  R_Date("OxA-19S/0178", 7730, 40)
  {
   Outlier("General", 0.05);
  };

  R_Date("OxA-30806", 7310, 40)
  {
   Outlier("General", 0.05);
  };
  Curve("=Marine20");
  Delta_R("Patella 101B",-114, 170);
  R_Date("OxA-34390", 7786, 39)
```

```
    {
     Outlier("General", 0.05);
    };
    Curve("=IntCal20");
    R_Date("OxA-30780", 7105, 40)
    {
     Outlier("General", 0.05);
    };
       Interval("Duration of Unit 101B");
       Date("Sample from Unit 101B");
   };
   Boundary("End Unit 101B");
Boundary("Start Unit 101A");
   Phase("Unit 101A")
   {
    Curve("=Marine20");
    Delta_R("Phorcus 101A",-257, 120);
    R_Date("OxA-28388", 7403, 33)
    {
     Outlier("General", 0.05);
    };
    Interval("Duration of Unit 101A");
    Date("Sample from Unit 101A");
   };
   Boundary("End Unit 101A");
Boundary("Start Unit 100");
   Phase("Unit 100")
   {
    Curve("=Marine20");
    Delta_R("Phorcus 100",-257, 120);
    R_Date("OxA-28387", 7212, 35)
    {
     Outlier("General", 0.05);
    };
```

```
  Curve("=IntCal20");
  R_Date("OxA-28397", 6772, 37)
  {
   Outlier("General", 0.05);
  };
  R_Date("OxA-30807", 6450, 40)
  {
   Outlier("General", 0.05);
  };
  R_Date("OxA-30779", 6447, 40)
  {
   Outlier("General", 0.05);
  };
      Interval("Duration of Unit 100");
      Date("Sample from Unit 100");
 };
 Boundary("End Unit 100");
 };
};
```

Supplementary References

1. Leorri E, Cearreta A, Milne G. Field observations and modelling of Holocene sea-level changes in the southern Bay of Biscay: implication for understanding current rates of relative sea-level change and vertical land motion along the Atlantic coast of SW Europe. *Quaternary Science Reviews* **42**, 59-73 (2012).

2. Gutiérrez Zugasti I, *et al.* Back to the Asturian: First result from the Mesolithic shell midden site of El Mazo (Asturian, Northern Spain). In: *Ancient Maritime Communities and the Relationship between People and Environment along the European Atlantic Coasts*. BAR Limited Internarcional Series 2570 (eds Daire, MY *et al.*). Archaeopress (2013).

3. Gutiérrez Zugasti I, González Morales MR. Intervención arqueológica en la cueva de El Mazo (Andrín, Llanes): campañas de 2009, 2010 y 2012. In: *Excavaciones arqueológicas en Asturias 2007-2012*). Gobierno del Principado de Asturias (2013).

4. Gutiérrez Zugasti I, *et al.* La ocupación de la costa durante el Mesolítico en el Oriente de Asturias: primeros resultados de las excavaciones en la cueva de El Mazo (Andrín, Llanes). *Archaeofauna* **23**, 25-38 (2014).

5. Gutiérrez Zugasti I, *et al.* Intervención Arqueológica en la cueva de El Mazo (Andrín, Llanes). Campañas de 2013, 2014, 2015 y 2016. In: *Excavaciones arqueológicas en Asturias 2013-2016*). Gobierno del Principado de Asturias (2018).

6. García-Escárzaga A. *Paleoclima y aprovechamiento de recursos costeros durante el Mesolítico en la región cantábrica (N de Iberia)*. BAR International Series, 2977, BAR Publishing (2020).

7. Simões, C. *The formation of Mesolithic shell middens in Atlantic Iberia. A geoarchaeological and micromorphological approach to the coastal adaptations of the Holocene hunter-gatherers*. Unpublished PhD dissertation, Universidad de Cantabria (2019).

8. Aldeias V, Bicho N. Embedded Behavior: Human Activities and the Construction of the Mesolithic Shellmound of Cabeço da Amoreira, Muge, Portugal. *Geoarchaeology* **31(6)**, 530-549 (2016).

9. Duarte C., Iriarte E, Diniz M, Arias P. The microstratigraphic record of human activities and formation processes at the Mesolithic shell midden of Poças de São Bento (Sado Valley, Portugal). *Archaeological and Anthropological Sciences* **11(2)**, 483-509 (2019).

10. Nesje A, Dahl SO. The Greenland 8200 cal. yr BP event detected in loss-on-ignition profiles in Norwegian lacustrine sediment sequences. *Journal of Quaternary Science* **16**, 155-166 (2001).

11. Rohling EJ, Pälike H. Centennial-scale climate cooling with a sudden cold event around 8,200 years ago. *Nature* **434**, 975 (2005).

12. Prasad S, Witt A, Kienel U, Dulski P, Bauer E, Yancheva G. The 8.2 ka event: Evidence for seasonal differences and the rate of climate change in western Europe. *Global and Planetary Change* **67**, 218-226 (2009).

13. Koutsikopoulos C, Le Cann B. Physical processes and hydrological structures related to the Bay of Biscay anchovy. *Scientia Marina* **60**, 9-19 (1996).

14. Durrieu de Madron X, Castaing P, Nyffeler F, Courp T. Slope transport of suspended particulate matter on the Aquitanian margin of the Bay of Biscay. *Deep Sea Research Part II: Topical Studies in Oceanography* **46**, 2003-2027 (1999).

15. Berger A. Long-Term Variations of Daily Insolation and Quaternary Climatic Changes. *Journal of the Atmospheric Sciences* **35**, 2362-2367 (1978).

16. Laskar J, Robutel P, Joutel F, Gastineau M, Correia ACM, Levrard B. A long-term numerical solution for the insolation quantities of the Earth. *A&A* **428**, 261-285 (2004).

17. Lorenz SJ, Kim J-H, Rimbu N, Schneider RR, Lohmann G. Orbitally driven insolation forcing on Holocene climate trends: Evidence from alkenone data and climate modeling. *Paleoceanography* **21** (2006).

18. Cionco RG, Soon WWH, Quaranta NE. On the calculation of latitudinal insolation gradients throughout the Holocene. *Advances in Space Research* **66**, 720-742 (2020).
19. Garcia-Soto C, Pingree RD, Valdés L. Navidad development in the southern Bay of Biscay: Climate change and swoddy structure from remote sensing and in situ measurements. *Journal of Geophysical Research: Oceans* **107**, 28-21-28-29 (2002).

20. Peliz Á, Dubert J, Santos AMP, Oliveira PB, Le Cann B. Winter upper ocean circulation in the Western Iberian Basin—Fronts, Eddies and Poleward Flows: an overview. *Deep Sea Research Part I: Oceanographic Research Papers* **52**, 621-646 (2005).

21. Le Cann B, Serpette A. Intense warm and saline upper ocean inflow in the southern Bay of Biscay in autumn–winter 2006–2007. *Continental Shelf Research* **29**, 1014-1025 (2009).

22. Garcia-Soto C, Pingree RD. Atlantic Multidecadal Oscillation (AMO) and sea surface temperature in the Bay of Biscay and adjacent regions. *Marine Biological Association of the United Kingdom Journal of the Marine Biological Association of the United Kingdom* **92**, 213 (2012).

23. Poppe GT, Goto Y. *European seashells. Vol. I (Polyplacophora, Caudofoveata, Solenogastra, Gastropoda)*. Verlag Christa Hemmen (1991).

24. Palacios N, Vega de la Torre JJ. *Guia de conchas de las playas y rías de Cantabria*. Gobierno de Cantabria (1997).

25. Gutiérrez Zugasti I. *La explotación de moluscos y otros recursos litorales en la región cantábrica durante el Pleistoceno final y el Holoceno inicial*. Publican, Ediciones Universidad de Cantabria (2009).

26. Bailey GN, Craighead AS. Late Pleistocene and Holocene coastal paleoeconomies: a reconsideration of the molluscan evidence from Northern Spain. *Geoarchaeology An International Journal* **18**, 175-204 (2003).

27. García-Escárzaga A. El Mesolítico Asturiense en el occidente de Cantabria: revisión de la información disponible a través de una reflexión crítica. *Kobie Serie Paleoantropología* **32**, 113-130 (2013).

28. Muñoz Fernández E, *et al.* Sondeo Arqueológico en el Abrigo de Barcenilla (T. M. de Piélagos, Cantabria). *Kobie Serie Paleoantropología* **32**, 79-112 (2013).

29. Álvarez-Fernández E, Álvarez-Alonso D, Cubas M, Cueto M. La cueva de El Pindal (Pimiango, Ribadedeva, Asturias): revisión de los materiales conservados en el Museo Arqueológico de Asturias. *Nailos* **2**, 191-210 (2015).

30. Bello-Alonso P, Ozkorta-Escribano L, Gutiérrez-Zugasti I. Un acercamiento al aprovechamiento de los recursos litorales durante el Mesolítico: los invertebrados marinos del abrigo de El Toral III (Llanes, Asturias). In: *La Investigación Arqueomalacológica en la Península Ibérica: Nuevas Aportaciones* (eds Gutiérrez Zugasti I, Cuenca Solana D, González Morales MR). Nadir Ediciones (2015).

31. Arias P, *et al.* Une nouvelle approche pour l'étude de l'habitat mésolithique dans le nord de la Péninsule Ibérique: Recherches dans le site au plein air d'El Alloru (Asturies, Espagne). *Séances de la Société Préhistorique Française* **6**, 159-190 (2016).

32. Fano MÁ. The Mesolithic "Asturian" culture (North Iberia), one century on. *Quaternary International* **515**, 159-175 (2019).

33. Rasmussen SO, Vinther BM, Clausen HB, Andersen KK. Early Holocene climate oscillations recorded in three Greenland ice cores. *Quaternary Science Reviews* **26**, 1907-1914 (2007).

34. Thomas ER, *et al.* The 8.2ka event from Greenland ice cores. *Quaternary Science Reviews* **26**, 70-81 (2007).

35. Domínguez-Villar, D. *et al.* Oxygen isotope precipitation anomaly in the North Atlantic region during the 8.2 ka event. *Geology* **37**, 1095-1098 (2009).

36. Moreno A, *et al.* Revealing the last 13,500 years of environmental history from the multiproxy record of a mountain lake (Lago Enol, northern Iberian Peninsula). *Journal of Paleolimnology* **46**, 327-349 (2011).

37. Stoll HM, *et al.* Paleoclimate and growth rates of speleothems in the northwestern Iberian Peninsula over the last two glacial cycles. *Quaternary Research* **80**, 284-290 (2013).

38. Smith AC, Wynn PM, Barker PA, Leng MJ, Noble SR, Tych W. North Atlantic forcing of moisture delivery to Europe throughout the Holocene. *Scientific reports* **6**, 24745 (2016).

39. Rossi C, Bajo P, Lozano RP, Hellstrom J. Younger Dryas to Early Holocene paleoclimate in Cantabria (N Spain): Constraints from speleothem Mg, annual fluorescence banding and stable isotope records. *Quaternary Science Reviews* **192**, 71-85 (2018).

40. Barber, D. C. *et al.* Forcing of the cold event of 8,200 years ago by catastrophic drainage of Laurentide lakes. *Nature* **400**, 344 (1999).

41. Clarke, G. K. C., Leverington, D. W., Teller, J. T. & Dyke, A. S. Paleohydraulics of the last outburst flood from glacial Lake Agassiz and the 8200BP cold event. *Quaternary Science Reviews* **23**, 389-407 (2004).

42. Wiersma, A. P. & Renssen, H. Model–data comparison for the 8.2kaBP event: confirmation of a forcing mechanism by catastrophic drainage of Laurentide Lakes. *Quaternary Science Reviews* **25**, 63-88 (2006).

43. LeGrande, A. *et al.* Consistent simulations of multiple proxy responses to an abrupt climate change event. *Proceedings of the National Academy of Sciences of the United States of America* **103**, 837-842 (2006).

44. Tindall, J. C. & Valdes, P. J. Modeling the 8.2ka event using a coupled atmosphere–ocean GCM. *Global and Planetary Change* **79**, 312-321 (2011).

45. Lewis, C. F. M., Miller, A. A. L., Levac, E., Piper, D. J. W. & Sonnichsen, G. V. Lake Agassiz outburst age and routing by Labrador Current and the 8.2 cal ka cold event. Quaternary International 260, 83-97 (2012).

46. Estrella-Martínez, J., Ascough, P. L., Schöne, B. R., Scourse, J. D. & Butler, P. G. 8.2 ka event North Sea hydrography determined by bivalve shell stable isotope geochemistry. *Scientific reports* **9**, 6753 (2019).

47. Mieszkowska N, Hawkins S, Burrows M, Kendall M. Long-term changes in the geographic distribution and population structures of *Osilinus lineatus* (Gastropoda: Trochidae) in Britain and Ireland. *Journal of the Marine Biological Association of the United Kingdom* **87**, 537-545 (2007).

48. Moore P, Hawkins S, Thompson R. Role of biological habitat amelioration in altering the relative responses of congeneric species to climate change. *Marine Ecology Progress Series* **334**, 11-19 (2007).

49. Hawkins SJ, *et al.* Complex interactions in a rapidly changing world: responses of rocky shore communities to recent climate change. *Climate Research* **37**, 123-133 (2008).

50. Mieszkowska N. Chapter 14 - Intertidal Indicators of Climate and Global Change. In: *Climate Change (Second Edition)* (ed Letcher TM). Elsevier (2016).

51. Nuñez S. *Dinámicas socio-ecológicas, resiliencia y vulnerabilidad en un paisaje atlántico montañoso: la región cantábrica durante el Holoceno*. Unpublished PhD dissertation, Universidad de Cantabria (2018).

52. Muñoz-Sobrino C, Ramil-Rego P, Gómez-Orellana L, Díaz Varela RA. Palynological data on major Holocene climatic events in NW Iberia. *Boreas* **34**, 381-400 (2005).

53. Gutiérrez Zugasti I. La biometría al servicio de la arqueomalacología: estrategias de recolección de moluscos en la región cantábrica entre el final del Paleolítico y los inicios del Neolítico. *Fervedes* **6**, 65-72 (2010).

54. Gutiérrez-Zugasti I. Coastal resource intensification across the Pleistocene–Holocene transition in Northern Spain: Evidence from shell size and age distributions of marine gastropods. *Quaternary International* **244**, 54-66 (2011).

55. Gutiérrez-Zugasti I, Tong E, García-Escárzaga A, Cuenca-Solana D, Bailey GN, González-Morales MR. Collection and consumption of echinoderms and crustaceans at the Mesolithic shell midden site of El Mazo (northern Iberia): Opportunistic behaviour or social strategy? *Quaternary International* **407**, 118-130 (2016).

56. García-Escárzaga A, Gutiérrez-Zugasti I, González-Morales MR, Cobo A. Shells and Humans: Molluscs and Other Coastal Resources from the Earliest Human Occupations at the Mesolithic Shell Midden of El Mazo (Asturias, Northern Spain). *Papers from the Institute of Archaeology* **27**, Art. 3 (2017).

57. García-Escárzaga A, *et al.* Stable oxygen isotope analysis of *Phorcus lineatus* (da Costa, 1778) as a proxy for foraging seasonality during the Mesolithic in northern Iberia. *Archaeological and Anthropological Sciences* **11**, 5631-5644 (2019).

58. Gutiérrez-Zugasti I, García-Escárzaga A, Martín-Chivelet J, González-Morales MR. Determination of sea surface temperatures using oxygen isotope ratios from *Phorcus lineatus* (Da Costa, 1778) in northern Spain: Implications for paleoclimate and archaeological studies. *The Holocene* **25**, 1002-1014 (2015).

59. García-Escárzaga A, Gutiérrez-Zugasti I, Schöne BR, Cobo A, Martín-Chivelet J, González-Morales MR. Growth patterns of the topshell Phorcus lineatus (da Costa, 1778) in northern Iberia deduced from shell sclerochronology. *Chemical Geology* **526**, 49-61 (2019).

60. Mannino MA, Spiro, BF, Thomas KD. Sampling shells for seasonality: oxygen isotope analysis on shell carbonates of the inter-tidal gastropod *Monodonta lineata* (da Costa) from populations across its modern range and from a Mesolithic site in southern Britain. *Journal of Archaeological Science* **30**, 667-679 (2003).

61. Soares AMM, Gutiérrez-Zugasti I, González-Morales M, Martins JMM, Cuenca-Solana D, Bailey GN. Marine Radiocarbon Reservoir Effect in Late Pleistocene and Early Holocene Coastal Waters off Northern Iberia. *Radiocarbon* **58**, 869-883 (2016).

62. García-Escárzaga A, et al. Bayesian estimates of marine radiocarbon reservoir effect in northern Iberia during Early and Middle Holocene. *Quaternary Geochronology* 67, 101232 (2022).

63. García-Escárzaga, A, Gutiérrez-Zugasti I. González-Morales M. Análisis arqueomalacológico de la unidad estratigráfica 108 del conchero mesolítico de El Mazo (Llanes, Asturias): conclusiones socio-económicas y metodológicas. In: *La Investigación Arqueomalacológica en la Península Ibérica: Nuevas Aportaciones* (eds Gutiérrez Zugasti I, Cuenca Solana D, González Morales MR). Nadir Ediciones (2015).
